# Advances and applications of brain organoids in central nervous system disorders: Bridging the gap from laboratory to clinic

**DOI:** 10.4103/NRR.NRR-D-24-01490

**Published:** 2025-06-19

**Authors:** Changle Fang, Qiulin Wang, Qiuxia Xiao, Xiaoxing Cai, Ruolan Du, Lulu Xue, Xiaohe Tian, Liulin Xiong

**Affiliations:** 1 Department of Anesthesiology, The Third Affiliated Hospital of Zunyi Medical University (The First People’s Hospital of Zunyi), Zunyi, Guizhou Province, China; 2 Institute of Neurosciences, Kunming Medical University, Kunming, Yunnan Province, China; 3 Institute of Neurological Disease, National-Local Joint Engineering Research Center of Translational Medicine, West China Hospital of Sichuan University, Chengdu, Sichuan Province, China; 4 State Key Laboratory of Biotherapy, Sichuan University, Chengdu, Sichuan Province, China

**Keywords:** acute brain injury, bioengineering, brain organoids, brain tissue transplantation, cerebral organoids, drug screening, induced pluripotent stem cell, neurodegenerative diseases, neurodevelopmental disorders

## Abstract

Investigating the mechanisms underlying central nervous system disorders is a major scientific issue in the 21^st^ century. However, the inaccessibility and complexity of the human brain have always represented a challenge in understanding the pathophysiology of the central nervous system. Brain organoids are self-assembled three-dimensional aggregates derived from pluripotent stem cells with cell types and structures similar to the embryonic human brain, giving them potential for investigating the atypical cellular, molecular, and genetic characteristics characteristic of central nervous system disorders. Brain organoids also provide a platform for drug screening and serve as a potential source for transplantation therapy for brain injuries. However, the broad application of brain organoids is hampered by several limitations, such as the lack of high-fidelity cell types, insufficient maturation, and considerable heterogeneity, undermining their reliability in specific applications. This review summarizes brain organoid evolution, discusses recent technological and methodological innovations, and reviews their applications in drug screening, transplantation therapy, and disease modeling, as well as clinical research progress. Additionally, we emphasize the limitations of current brain organoid research and explore the potential for advancing the technology to enhance its applicability.

## Introduction

The central nervous system (CNS), comprising the brain and spinal cord, serves as the body’s primary communication center (Rustenhoven and Kipnis, 2022). CNS diseases encompass a wide range of conditions, such as nervous system injuries (Feng et al., 2024), cerebrovascular disease (Feng et al., 2024), neurodegenerative diseases (Li and Wang, 2023), headache disorders (Ashina et al., 2021), demyelinating diseases (Huang et al., 2024a), congenital conditions (such as microcephaly, megalocephaly, and focal cortical dysplasia) (Almacellas Barbanoj et al., 2024; Goel et al., 2024; Li et al., 2024), neurodevelopmental disorders (van Eyk et al., 2023), and infectious brain diseases (Priyathilaka et al., 2022). Because of ethical concerns and the challenges in accessing brain tissues, for centuries, CNS disease research relied on *in vitro* cell models, animal models, and post-mortem brain tissues (Kawakita et al., 2022). These traditional methods have provided valuable mechanistic insights. However, they involve several inherent limitations, including the oversimplification of *in vitro* cell cultures, the limited accuracy of animal models of diseases, and the ethical concerns associated with post-mortem specimens (Buonfiglioli et al., 2025).

Brain organoids are stem cell–derived three-dimensional (3D) structures that can replicate many critical aspects of brain development (Park et al., 2023; Zhou et al., 2023b). They effectively model brain developmental processes and provide a platform for investigating neurological disorders, including neurodevelopmental and neurodegenerative diseases (Li et al., 2023). Furthermore, brain organoids can simulate the brain microenvironment (Qu et al., 2023), facilitating the study of synaptic connections and cell-to-cell interactions in a more physiologically relevant context (Revah et al., 2022).

Recent studies have focused on developing brain organoids that more accurately represent real-world conditions, such as those with high-fidelity cells (Andrews and Kriegstein, 2022; Velasco, 2022). High-fidelity cells closely resemble real brain tissue cells in both morphological and functional characteristics, including properly differentiated glial lineages, mature and functional glutamatergic and GABAergic neurons, and cells capable of forming complex network structures similar to the cerebral cortex.

This review introduces the concept of brain organoids and their historical evolution, and discusses the latest relevant biotechnical innovations. Next, we summarize the current applications of brain organoids, such as platforms for drug screening and neurotoxicity testing, cell transplantation therapy, and CNS disease models, and address their limitations. In conclusion, this review emphasizes the potential of brain organoids to drive progress in CNS disease research and deepen our comprehension of their underlying mechanisms.

## Retrieval Strategy

The literature cited in this review is mainly from SCOPUS, PubMed, and Web of Science, and the publication time is up to March 30, 2025. The medical subject headings (MeSH) or keywords are used in the search, including “organoids,” “cerebral organoids,” “brain organoids,” “central nervous system diseases,” “organoid culture,” “biomedical engineering,” “microfluidics,” “organoids-on-chip,” “bioprinting,” “vascularization,” “neuroglia,” “microglia,” “astrocyte,” “oligodendrocyte,” “assembloids,” “genetic engineering,” “CRISPR/Cas9,” “Drug screening,” “Zika virus,” “neurotoxicology,” “transplantation,” “brain injuries,” “brain injuries, traumatic,” “hypoxia-ischemia, the brain,” “ischemic stroke,” “neurodevelopmental disorders,” “autism spectrum disorder,” “Down syndrome,” “microcephaly,” “Timothy syndrome,” “neurodegenerative disorders,” “Alzheimer disease,” “Parkinson disease,” “Huntington disease,” “psychiatric disorders,” “schizophrenia,” “bipolar disorder,” “brain neoplasms,” and “ethics.” After eliminating irrelevant studies from the results, the abstracts of each article were reviewed, and studies with low relevance were discarded. Finally, the full texts of filtered articles were read carefully, and studies with clear experimental protocols were included.

## Overview of Brain Organoids

**Figure 1:**
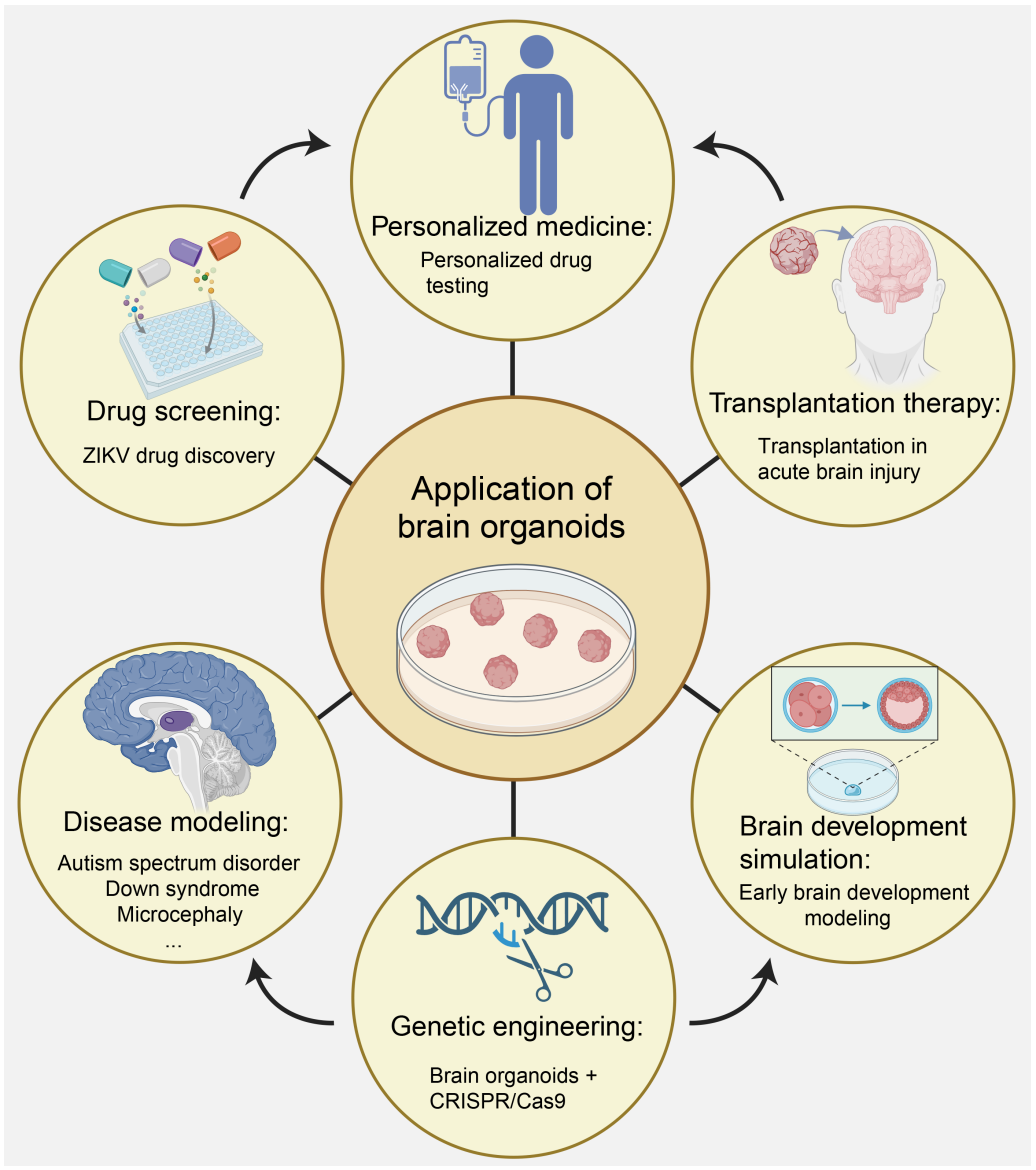
Applications of brain organoids.

Organoids are groups of cells that proliferate in a 3D structure and have the ability to regenerate themselves and differentiate into multiple functional cell types. Organoids can therefore mimic the functions and cell types of human organs (Sun et al., 2022). In 2013, 3D brain organoids were used as an *in vitro* model of the development of human brains and microcephaly (Lancaster et al., 2013). Although several aspects require further improvement, brain organoids have already been widely applied in neuroscience, including neurological disease modeling, neurodevelopmental simulation, drug screening, transplantation therapy, and potential clinical applications (**Figure 1**), demonstrating the enormous potential of brain organoids in treating nervous system disorders.

### Memorabilia of the development of brain organoids

**Figure 2:**
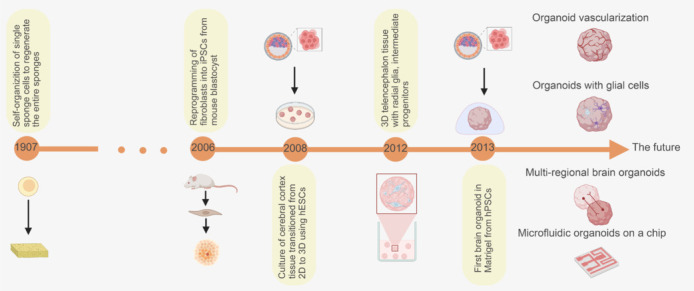
Milestones of brain organoids. hESCs: Human embryonic stem cells; hPSCs: human pluripotent stem cells; iPSCs: induced pluripotent stem cells.

Organoids are primarily derived from human pluripotent stem cells (hPSCs), such as human induced pluripotent stem cells (iPSCs) and human embryonic stem cells (hESCs). This highlights the significance of stem cell technology in organoid research. In 1907, Wilson demonstrated that free sponge cells could regenerate entire sponges, marking the first description of organismal regeneration *in vitro*. Stem cell technology continued to advance over the following century. The reprogramming of fibroblasts into iPSCs in 2006 revolutionized stem cell and organoid research. In 2008, the introduction of 3D aggregate culture methods marked the transition of organoid research from 2D to 3D, enabling hESCs to generate cerebral cortical tissue (Eiraku et al., 2008). In 2012, researchers first successfully generated 3D telencephalic tissues containing radial glial cells and intermediate progenitor cells from human iPSCs. These iPSC-derived multilayered structures expressed characteristic embryonic telencephalic gene profiles (Mariani et al., 2012). Lancaster’s milestone 2013 study demonstrated Matrigel-encapsulated hPSC-derived neural tissues using orbital shaking for optimized nutrient perfusion (Lancaster et al., 2013). The resulting aggregates, termed brain organoids, effectively simulated early human neurodevelopmental processes. Recent efforts have focused on developing more physiologically relevant brain organoids, including vascularized organoids, organoids with glial cells, multiregional assembloids, and organoid-on-a-chip microfluidic platforms. Organoids have emerged as a rapidly growing research area over the last decade, exhibiting an accelerating trend as illustrated in **Figure 2**.

### Culturing of brain organoids

**Figure 3:**
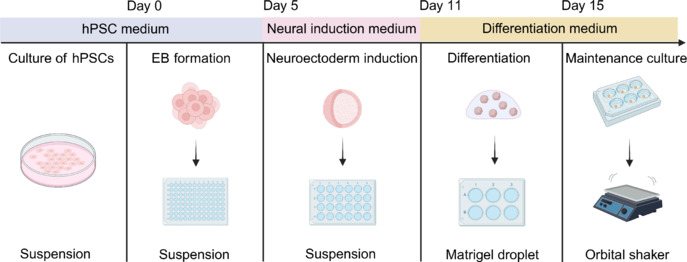
Process of cultivating brain organoids. EB: Embryoid body; hPSCs: human pluripotent stem cells.

The traditional approach to organoid formation involves the 3D culture of hPSCs in the presence of growth factors and small molecules. These cells proliferate and self-organize into 3D organoid structures. Organoids generated using this method can be passaged and cryopreserved for future applications (**Figure 3**; Lancaster et al., 2013, 2017; Lancaster and Knoblich, 2014; Yakoub and Sadek, 2018; Velasco et al., 2019). The generation of brain organoids primarily involves hPSC culture, embryoid bodies (EBs) formation, neuroectoderm induction, differentiation, and maintenance culture (Lancaster et al., 2013; Lancaster and Knoblich, 2014). hPSCs are first plated on mouse embryonic fibroblast feeder layers, regularly passaged, and monitored to assess their readiness for EB formation (Watanabe et al., 2007). Subsequently, hPSCs are transferred to hESC medium supplemented with low concentrations of essential fibroblast growth factor and Rho-associated protein kinase inhibitors and seeded in low-adherence 96-well plates for EB formation. On day 5, the EBs become translucent with smooth edges and are transferred to a low-adherence 24-well plate. Neural induction medium is then used to promote neuroectoderm formation. These neuroectodermal progenitor cells spontaneously organize into multiple neural rosette 3D structures, which resemble the apical lumen of neural tube neuroepithelium (Lancaster et al., 2013). On day 11, qualified neuroectodermal progenitor cells are embedded into Matrigel. These embedded cells are transferred to a differentiation medium for a 4-day static culture. On day 15, the organoids are transferred to a rotating bioreactor or orbital shaker containing a differentiation medium. The organoids typically reach a size of 2–4 mm after approximately 2 months. Region-specific brain organoids have been developed, which facilitate physiological and pathological studies of brain development.

## Biotechnical Innovations for Brain Organoids

Although the prospect of 3D brain organoids is exciting, the field faces the following challenges: (1) the high heterogeneity of the brain organoids (Jiang et al., 2020; Zhou et al., 2023a); (2) the lack of a functional circulatory system to transport nutrients and oxygen, causing cell death and constraining the size of brain organoids (Werschler and Penninger, 2023); (3) the ability to effectively investigate brain circuitry and function is limited (Chiola et al., 2022; Pigoni et al., 2023); and (4) the absence of glial cells such as astrocytes and microglia (de Majo et al., 2023; Han et al., 2023). Therefore, while organoids have great potential for application in CNS disorders, several significant obstacles must be addressed. Reassuringly, recent bioengineering innovations are overcoming these challenges to realize the potential of brain organoids.

### Microfluidic organoids-on-a-chip

Microfluidics is the technology of manipulating and controlling small amounts of fluids at a microscopic scale (Shakeri et al., 2023). Microfluidic devices use precisely designed channels, valves, and other microstructures to enable fluid delivery, mixing, separation, and other operations (Palasantzas et al., 2023). Organoids-on-chips is a high-throughput *in vitro* model construction platform created by combining organoids and organ-chips technologies (Castiglione et al., 2022; Wang et al., 2024c). The physiological microsystems of human organoids built on microfluidic chips offer advantages in terms of controllability and modeling standardization and can achieve more complex model construction through co-culture techniques (Busek et al., 2023; Zeng et al., 2023).

Microfluidic organoids-on-a-chip co-culture systems offer the potential to mimic physiological environments through controlled fluid dynamics and nutrient delivery (Duzagac et al., 2021; Nolan et al., 2023). Recently, microfluidic co-culture systems have been used for brain organoid culture to more accurately replicate the *in vivo* environment under *in vitro* conditions (Ao et al., 2022). Polydimethylsiloxane is the most frequently utilized material in cell biology applications owing to its chemical inertness, mechanical robustness, biocompatibility, optical clarity, gas permeability, and cost-effectiveness (Nge et al., 2013). Two primary methods are used to introduce fluid into the chip channels—static and dynamic culture systems.

Brain organoids typically exhibit heterogeneity in their developmental stages because they form through the spontaneous self-organization of stem cells (Tang et al., 2022). Furthermore, manual manipulation during cultivation exacerbates their heterogeneity, hindering the production of consistent organoids and reducing the reliability of experimental outcomes. Additionally, the labor-intensive nature of this method presents challenges for large-scale production requirements, such as drug screening and neurotoxicity testing (Zhao et al., 2024; Hu et al., 2025).

The static culture system aims to standardize brain organoid cultivation, reduce costs, and simplify operation workflows. In this system, brain organoids are usually placed in an environment without external forces—a space-saving and practical microfluidic device. In this environment, nutrients and metabolic waste are exchanged with the medium by diffusion. Renner et al. (2020) reported an automated system that reliably produces human midbrain organoids in a 96-well plate format. This device minimizes batch variances by eliminating the Matrigel embedding step and mitigating paracrine signaling, which may affect adjacent organoids. Cho et al. (2021) used a micropillar array to generate brain organoids from iPSCs. This device allows individual cells to form embryoid bodies before neural induction and differentiation, which facilitates the in situ assembly of brain organoids. This method simplifies procedural steps, reduces the risk of cell contamination in experiments, and improves the efficiency of brain organoid production.

The dynamic culture system mimics more realistic *in vivo* physiological conditions by actively emulating the vascular system using mechanical pumps. This system provides a more complex and controllable environment, enhancing versatility and precision compared to the static culture system. A six-well chip with a mechanical pump microfluidic system was reported to culture midbrain organoids. This device ensures a continuous laminar flow for medium delivery, which improves the oxygen levels at the organoids’ inner layers, significantly reduces the necrotic core, and promotes the differentiation of neural stem cells into dopaminergic neurons (Berger et al., 2018; Babu et al., 2024). Although the pump-based dynamic culture systems more accurately replicate the *in vivo* environment, they also increase system complexity and cost. To address these challenges, researchers have developed techniques to simplify the cultivation system while maintaining a constant flow within the microfluidic chip. For example, Cho et al. (2021) developed a novel chip containing five chambers (three for medium storage and two for brain organoid culture), facilitating periodic fluid flow through a bidirectional rocker that utilizes hydrostatic pressure generated by medium-level differences between chambers connected by microchannels. This device eliminates the requirement for external tubing and driving pumps. Organoids cultured in this microfluidic chip exhibited enhanced cell proliferation and reduced cell death. Pump-free microfluidic culture platforms can significantly improve the culture conditions for brain organoids.

In summary, microfluidic organoids-on-a-chip significantly enhance the culture conditions of brain organoids. Static culture systems can improve the reproducibility and consistency of brain organoid cultures, making it possible to produce brain organoids on a large scale, providing a foundation for high-throughput drug screening. Dynamic culture systems offer a more sufficient nutrient supply and waste removal mechanisms, allowing for more mature brain organoids, which better simulate the human internal environment and are more suitable for long-term culture. However, current microfluidic organoids-on-a-chip still face several limitations. For example, in the absence of nutrient supply and waste removal mechanisms, the static culture system may lead to malnutrition and metabolite accumulation in the brain organoids’ inner layers, adversely affecting their health and function. This also means that the static culture system is not suitable for the long-term culture of brain organoids. Dynamic culture systems include complete nutrient supply and waste removal mechanisms, but the complexity of manufacturing these systems requires advanced technology and professional knowledge, constituting a barrier for some researchers. The costs associated with establishing and maintaining these systems are very high, making it challenging to achieve large-scale production (**Additional Table 1**). With advances in automation technology, it is expected that higher-throughput and lower-cost culture solutions will be adopted, which will enable microfluidic organoids-on-a-chip technology to fully realize its potential.

**Additional Table 1:** Comparisons of the static and dynamic culture systems

Models	Benefits	Limitations
The static culture systems	• Low equipment dependence and high operability	• Lack of nutrient supply and waste removal mechanisms
	• High reproducibility and consistency	• Difficult for long-term cultivation
	• Cost-effective and time-saving	• Cannot simulate dynamic physiological conditions
	• Suitable for large-scale production	
The dynamic culture systems	• High maturation	• High system complexity and equipment dependence
	• Suitable for long-term cultivation	
	• Narrowing the gap with *in vivo* states	• Considerable financial cost

### 3D bioprinting of brain organoids

3D bioprinting is a novel technology that combines 3D printing with biomaterials, including cells and biological materials (Ju and Dong, 2024; Zhu et al., 2025). Researchers employ various printing methods, such as laser-assisted inkjet- and extrusion-based bioprinting, to deposit cells and biomaterials layer by layer, creating 3D biological structures that mimic natural forms and functions (Jiu et al., 2024; Yan et al., 2024). This technology enables researchers to fabricate complex biological structures with high precision (Maharjan et al., 2024). Currently, it is extensively applied in tissue engineering for bones (Bai et al., 2024), pancreas (Salg et al., 2022), skin (Lorenzetti et al., 2024), liver (Sun et al., 2024a), and brain (Zamponi et al., 2023).

Compared to conventional organoid culture methods, organoid bioprinting offers several advantages. For instance, before organoid culture, a 3D bioprinter can be used to construct specialized culture plates, which are often combined with microfluidic systems (Wu et al., 2023b). High-density bioprinting using cell-laden bioinks can reduce manual handling and facilitate organoid maturation (Wu et al., 2023b). The manual creation of tiny droplets to embed live cells or embryoids into scaffolds often results in uneven droplet sizes, human errors, or improper handling procedures. Bioprinters that can accommodate thermosensitive biomaterials simplify this process, reducing inappropriate handling and thus decreasing experimental time. Printing various cell types in specific spatial arrangements can generate hierarchical structures and vascular network conformations with diverse cell interactions, more accurately replicating the complex interactions occurring *in vivo*. Additionally, automated bioprinting can establish a reproducible high-throughput organoid production platform, thereby enhancing drug screening development (Chliara et al., 2022; Tebon et al., 2023; Dai et al., 2024).

3D printing technology has been effectively applied to brain organoids research (Rezaei et al., 2023; Xu et al., 2024). For example, Reid et al. (2016) used a modified injector and an extrusion-based 3D printer to successfully print iPSCs into Geltrex. They also demonstrated that injection into a differentiation-conducive environment promoted embryoid differentiation and sustained iPSC growth. Tomaskovic-Crook et al. (2023) employed a 3D bioprinter to produce brain organoids, in which a prepolymer solution of gelatin methacrylate (GelMA) was loaded into the syringe barrel of the printer and then extruded onto a porous array with a piston. Finally, iPSCs were seeded onto the GelMA layer to initiate organoid formation. This approach enables iPSCs to form organoids at the original location after seeding on GelMA, which minimizes further disruption of cells and organoids. Zhang et al. (2022) described bioprinting Alzheimer’s disease (AD) organoids using human neural progenitor cells with 2% Matrigel and 2% alginate as bioinks. This approach more accurately replicated the tissue microenvironment and detected elevated Aβ protein levels and Aβ and tau gene expression, which are commonly linked to AD progression. Roth et al. (2023) developed a spatially patterned organoid transfer platform comprising a magnetized 3D printer and hydrogels loaded with iron oxide nanoparticles to achieve the deposition, transport, and spatial positioning of brain organoids. Using this platform, they created precisely arranged assembloids composed of patient-derived glioblastoma organoids and iPSC-derived neural organoids. These neural assembloids, created by this platform, can serve as *in vitro* models for studying neurodevelopmental events and the progression of neurological diseases.

Although the 3D bioprinting of organoids shows great promise, several challenges remain unsolved. One of the main obstacles to overcome is the development of universal bioinks (Wang et al., 2024b). Bioinks serve as scaffold materials for cells in 3D bioprinting, typically consisting of hydrogels, culture media, growth factors, and other components. Natural biomaterials, such as alginate, demonstrate excellent biocompatibility but possess loose network structures, typically leading to poor mechanical performance and high swelling rates (Zhang et al., 2015; He et al., 2022). Synthetic materials, such as GelMA and polyethylene glycol diacrylate, provide adjustable mechanical properties, ensuring excellent printability and shape fidelity, but they require ultraviolet ray crosslinking, which may damage organoids (Kratochvil et al., 2019; Wang et al., 2024d). Consequently, it is necessary to develop synthetic bioinks with excellent mechanical and biological functions. Additionally, acquiring 3D bioprinting equipment can be costly, especially when specific features (e.g., control systems for thermosensitive biomaterials) are needed (Ding et al., 2023). It is challenging to balance the long-term advantages of creating more precise organoids with the significant financial costs. Therefore, creating more cost-efficient 3D bioprinting equipment poses a major challenge.

### Vascularization of brain organoids

Under normal physiological conditions, the vascular and nervous systems of the human brain develop synergistically (Lénárt et al., 2024). 3D brain organoids generate complex neural networks, but these structures lack vascular systems, resulting in slow nutrient transport, waste elimination failure, and insufficient oxygenation, which limits the size and maturation of brain organoids. Early studies typically used an orbital shaker (Nickl et al., 2023) and organotypic brain slice culture (Kelava et al., 2022) to address these questions, but these methods have obvious drawbacks. For example, orbital shakers can only improve oxygen and nutrient supply to the surface, resulting in cell death in the brain organoids’ innermost layers (Huang et al., 2023; Peng et al., 2023). Human organotypic brain slice cultures can deliver oxygen and nutrients to deeper regions, but the repeated slicing damages the specific 3D structures (Bak et al., 2024). Given that the neurovascular network plays a crucial role in normal brain development and function, vascularization is essential for developing accurate human brain models.

Currently, the vascularization of brain organoids is primarily achieved using two approaches: *in vivo* and *in vitro* cultivation. One method for *in vitro* culture involves co-culturing induced brain organoids with vascular cells during the early developmental stages. Sun et al. (2022) generated vascular and brain organoids from different sources, encapsulating both organoid types in Matrigel on day 12 for fusion culture, resulting in brain organoids with a vascular system. The fused brain organoids exhibited a robust vascular network-like structure and demonstrated an increase in neural progenitor cells, which is consistent with the possibility of the vascular regulation of neural development. Another study used iPSCs from the same source to induce brain organoids and endothelial cells (ECs), embedding the organoids in Matrigel-containing ECs on day 34, successfully achieving organoid vascularization (Shaji et al., 2024). Similar vascularization strategies have been extensively reported. For instance, Ahn et al. (2021) produced vascularized human cortical organoids by co-culturing cortical organoids with human vascular organoids. Shi et al. (2020) co-cultured brain organoids with human umbilical vein endothelial cells, which formed tubular structures within the brain organoids on day 42, promoting neurogenesis and blood morphogenesis. Notably, studies have shown that these vascularized organoids contain more active neurons than traditional non-vascularized brain organoids, accelerating neurogenesis and maturation in human brain organoids (Wang et al., 2021b; Sun et al., 2022).

Another method for generating vascularized brain organoids *in vitro* is to treat brain organoids with vascular developmental factors. For example, Cakir and colleagues found that ETV2 in brain organoids triggered EC formation. The overexpression of ETV2 induces vascular-like structures, enhances oxygen diffusion, and promotes brain organoid growth and neuron maturation (Cakir et al., 2019). Similarly, Ham et al. (2020) treated brain organoids with Wnt7a and VEGF in the early stages and observed the formation of outer layers composed of pericyte-like cells, which contribute to the development and maintenance of the blood–brain barrier (BBB).

*In vivo* cultivation is a technique whereby brain organoids are transplanted into a living organism, leveraging the host’s circulatory system to continuously supply oxygen and nutrients to the organoids (Li et al., 2022a). This approach establishes a theoretical framework for the potential therapeutic applications of brain organoid transplantation. For instance, Mansour et al. (2018) cultured organoids *in vitro* for 40–50 days and then transplanted them into a cavity created in the retrosplenial cortex of immunodeficient mice. The findings indicated that the majority of the transplanted organoids persisted for more than 180 days. A separate study involved implanting human brain organoids into the brains of mice. Within 14 days post-transplantation, the blood vessels had significantly attached to human brain organoids, with 85.4% of the transplanted organoids successfully vascularizing. Those that failed to vascularize did not survive, indicating that transplanted organoids rely on blood circulation for oxygen and nutrient delivery to sustain survival (Wang et al., 2025).

In recent years, remarkable progress has been made in vascularizing human brain organoids. However, the full potential has not yet been fully realized, and these innovations remain in the early stages. Several significant challenges must be addressed to attain complete vascularization of brain organoids. First, brain organoids currently lack an adequate pumping mechanism for blood circulation. Second, the absence of blood cells within brain organoids *in vitro* poses a barrier, not only hindering the development of brain organoids but also making it impossible to investigate whether blood cells are crucial for brain development. Furthermore, the current vascularized brain organoids exhibit very limited properties of the BBB, which may impair their growth and restrict their application to simulating CNS disorders. In addition, brain organoids at present lack the secretion of cerebrospinal fluid (CSF) from the choroid plexus and the blood–CSF barrier. Future research that combines vascularized brain organoids with choroid plexus organoids could represent a strategy to generate a more complete vascular system in brain organoids.

### Brain organoids with glial cells

In healthy individuals’ brains, glial cells such as microglia, astrocytes, and oligodendrocytes coexist with neurons and play critical roles in brain development and disease progression. However, brain organoids are currently unable to produce mature oligodendrocytes and microglia. Instead, they mainly comprise certain neural progenitor cells, mature neuronal subtypes, astrocytes, and oligodendrocyte progenitor cells (Tidball et al., 2023). To address this limitation, researchers have begun to develop brain organoids containing glial cells.

Several studies have proposed methods for enriching oligodendrocytes in brain organoids. For example, Madhavan et al. (2018) and Marton et al. (2019) promoted the maturation of oligodendrocytes in brain organoids by using cytokines that enhance the survival, proliferation, and differentiation of oligodendrocyte progenitor cells, such as platelet-derived growth factor, insulin growth factor-1, and triiodothyronine. The results revealed that brain organoids exhibit mature oligodendrocytes adjacent to neurons and astrocytes, which generate myelin sheaths. Monzel et al. (2017) used a similar approach to generate human midbrain organoids from iPSC-derived neuroepithelial stem cells. These brain organoids exhibit mature oligodendrocytes with myelin sheaths and characteristic nodes similar to the structure of Ranvier.

As innate immune cells of the CNS, microglia interact with neurons, oligodendrocytes, and astrocytes (Fattorelli et al., 2021) and play an important role in maintaining brain homeostasis and immune defense (Thion et al., 2018). Therefore, microglia are essential components of functionally accurate brain models (Ning et al., 2025; Yang et al., 2025). Microglia originate from progenitor cells in the yolk sac, which is derived from the mesoderm, and follow a unique developmental trajectory. Consequently, microglia do not form in patterned brain organoids derived from the neuroectoderm (Prinz et al., 2021). Current methods for creating brain organoids containing microglia typically involve producing the two separately, followed by co-culturing them rather than directly inducing microglia within the brain organoids. For example, Sabate-Soler et al. (2022) cultured iPSC-derived midbrain organoids and macrophage precursor cells separately and initiated co-culture on day 15 of dopaminergic differentiation, demonstrating that these organoids expressed microglia-specific markers and mediated normal phagocytosis. Abreu et al. (2018) co-cultured iPSC-derived brain organoids with immortalized human microglia (SV40). When microglia-co-cultured brain organoids were treated with lipopolysaccharide and infected with a flavivirus, increased inflammatory cytokines and gene expression levels were observed, indicating that these brain organoids could produce an inflammatory response to infectious diseases. Popova et al. (2021) co-cultured iPSC-derived microglia with 5-week-old cortical brain organoids. The findings revealed decreased cellular stress and interferon expression in brain organoids co-cultured with microglia, suggesting that microglia promoted synaptic remodeling and neural network maturation in the brain organoids.

Co-culturing brain organoids with microglia offers a more physiologically relevant brain environment. However, there is still no established standard for how and when to introduce iPSC-derived microglia during brain organoid development, and additional experiments are required.

### Application of multiregional brain organoids

The development of the normal brain and the manifestation of its functions, as well as the pathological mechanisms underlying most neurological disorders, involve circuit transmission between different brain regions (Qiu et al., 2022). Region-specific brain organoids represent one key approach to investigating these critical issues. Various regionally fused brain organoids have been developed to study the formation of neural circuits across different brain regions and neuron–neuron interactions.

During brain development, the formation of brain regions is predominantly controlled by the orchestrated activities of morphogens, including wingless-related integration site (Aoki et al., 2024), sonic hedgehog (Douceau et al., 2023), and basic fibroblast growth factor (Numakawa and Kajihara, 2023). These molecules act along two primary developmental axes: the rostral-caudal axis (anterior to posterior) and the dorsal-ventral axis (dorsal to ventral). During organoid induction, treatment with morphogens or their small-molecule equivalents produces regionally fused brain organoids. For example, Pavon et al. (2024) used a novel gradient diffusion device to generate spatially patterned forebrain organoids, which reliably produces sonic hedgehog gradients to establish the dorsal–ventral axis and promotes regionalization from the forebrain cortex to the basal ganglia along this axis. Xue et al. (2024) simulated morphogen concentration gradients using a microfluidic device, creating rostral–caudal and dorsal–ventral axes, ultimately constructing a microfluidic NT-like structure. This facilitated the regionalization of the rostral–caudal pattern from the forebrain and midbrain to the hindbrain. These methods provide proof-of-concept for generating multi-regional neural tissues from hPSCs by controlling morphogen gradients.

An alternative strategy to creating multi-regional brain organoids from hPSCs involves assembling multiple organoids from distinct brain regions. During cortical development, excitatory glutamatergic neurons originating from dorsal forebrain progenitors and inhibitory GABAergic interneurons from ventral forebrain progenitors integrate to form cortical neural circuits. Studies have demonstrated that fusing dorsal and ventral forebrain organoids recapitulates the migration of GABAergic neurons from the ventral to the dorsal forebrain (Bagley et al., 2017; Birey et al., 2022). Similarly, fused brain organoid models created by this method have been widely used to study the formation of neural networks and circuits, such as striatum–midbrain assembloids (Ozgun et al., 2024), cortex–striatum assembloids (Miura et al., 2020), striatum–substantia nigra assembloids (Wu et al., 2024b), and thalamus–cortex assembloids (Patton et al., 2024). Additionally, fused brain organoid structures are increasing in complexity, such as the multi-regional assembloids generated by Andersen et al. (2020), which integrate three types of organoids (including the cerebral cortex, spinal cord, and skeletal muscle). These fused organoids exhibit muscle contraction in response to optogenetic stimulation of cortical neurons via motor neurons. Reumann et al. (2023) produced spatially organized ventral–midbrain–striatum–cortical organoids from hPSCs to investigate the functional innervation of dopaminergic circuits in the striatum and cortex.

Although efforts to recreate human brain characteristics through organoid fusion have made advances, significant challenges remain, such as achieving precise control over organoid fusion and addressing the low reproducibility of assembloids. Future advancements in multi-regional brain assembloids and organoid fusion techniques are expected to provide more accurate brain models for studying brain development, neural circuit formation, and neurological disease phenotypes.

### Genetic engineering in brain organoids

A variety of genetic engineering techniques are being employed with brain organoids. These methods facilitate precise modifications of the genomic DNA sequence (Ji et al., 2023). Clustered regularly interspaced short palindromic repeats/CRISPR-associated protein 9 (CRISPR/Cas9) technology is the most widely used genome editing tool. CRISPR-based studies on neurological diseases typically utilize immortalized human cell lines or non-human cells (Chen et al., 2017; Wang et al., 2021c; Tian et al., 2022). This research has successfully identified numerous potential mechanisms associated with neurological diseases, but they are subject to significant limitations. For instance, employing non-human cells or transformed cell lines for modeling may overlook many human neuron-specific genes (Swingler et al., 2023). Using human brain organoids combined with CRISPR/Cas9 technology provides an opportunity to investigate neurological diseases in a human tissue-like environment. For example, Tang et al. used iPSCs derived from Down syndrome (DS) patients to generate DS brain organoids and employed CRISPR/Cas9 and CRISPR interference (CRISPRi) to suppress the DSCAM/PAK1 pathway, which exhibits heightened activity in DS. The findings demonstrated that this intervention reversed abnormal neurogenesis, increasing the size of organoids derived from DS iPSCs (Tang et al., 2021).

In summary, these results highlight the significant utility of CRISPR in disease modeling and therapeutic testing, facilitated by disease-specific neural organoids and their isogenic controls.

## Applications in Drug Screening and Neurotoxicity Assessment

Another major application of brain organoids is drug screening and neurotoxicity assessment. The conventional drug discovery process begins with the screening of compounds and targets. Subsequently, the efficacy and toxicity of candidate drugs are validated using animal models. Clinical trials are conducted to test the drugs in humans, based on the results from preclinical studies. Ultimately, successful drugs are brought to market. However, due to substantial differences between animal models and the human nervous system, numerous therapies that succeed in preclinical trials fail in subsequent clinical studies (Mulder et al., 2023). iPSC-derived brain organoids enable both *in vitro* and *in vivo* research, offering a relevant innovation for modeling the physiological mechanisms of the human brain (Sabogal-Guaqueta et al., 2024). Preclinical research based on brain organoids allows for a comprehensive evaluation of the effects of drugs or toxins on brain progenitor cell proliferation, neuronal differentiation, migration, and neurogenesis, providing more reliable data for clinical trials and final regulatory approval (Dixon and Muotri, 2023; Antón-Bolaños et al., 2024).

### Drug screening

Despite the rapid progress in preclinical drug development, it can still take considerable time to assess the efficacy and safety of drugs in humans. More effective and large-scale screening of small-molecule therapies is therefore urgently required. For instance, brain organoids with Zika virus (ZIKV) infection have been utilized to facilitate drug screening. ZIKV is a single-stranded RNA virus transmitted by mosquito vectors. A prominent feature of ZIKV infection is microcephaly, characterized by an abnormally small head circumference (Zhang et al., 2024a), with a large number of ZIKV-associated microcephaly cases reported during the 2015/16 ZIKV epidemic in South America (Lieberthal et al., 2024). Given the urgency of this public health issue, rapid *in vitro* screening for anti-ZIKV compounds using brain organoids is crucial. For instance, Xu et al. (2016) employed a high-throughput drug screening on the brain organoid platform to target ZIKV from 6000 compounds, including pharmacologically active compounds, clinical candidates, and approved drugs. This method led to the identification of several potential anti-ZIKV compounds, finding that combining various compounds could further protect neurons and glial cells from ZIKV-induced cell death (Xu et al., 2016). Another high-content chemical screen for anti-ZIKV activity in human forebrain organoids identified that emetine dihydrochloride hydrate and amodiaquine dihydrochloride dihydrate were effective in inhibiting ZIKV infection (Zhou et al., 2017). In addition, brain organoid models also play a crucial role in developing drugs against severe acute respiratory syndrome coronavirus 2 (SARS-COV-2). Some researchers have used human brain organoids to screen for therapeutic strategies targeting SARS-CoV-2 infection. For example, Song et al. (2021) demonstrated that blocking angiotensin converting enzyme 2 (ACE2) receptors in human brain organoids using anti-ACE2 antibodies and anti-spike antibodies collected from the CSF of Coronavirus disease 2019 (COVID-19) patients effectively inhibited SARS-CoV-2 neuroinvasion. Wang et al. (2021a) reduced SARS-CoV-2 infection and rescued virus-induced neuron death in iPSC-derived neurons and astrocytes using treatment with the antiviral agent remdesivir. The Food and Drug Administration (FDA)-approved antiviral agent Sofosbuvir has also been shown to inhibit SARS-CoV-2 replication and rescue these neuronal alterations in infected brain organoids (Mesci et al., 2022). These results provide a theoretical basis for anti-SARS-CoV-2 drugs.

With the emergence of high-throughput, standardized brain organoid production platforms, enhancing the correlation between preclinical brain organoid studies and clinical trials is crucial for improving the efficiency and success rate of drug development. First, it is necessary to verify the reliability of brain organoid models, such as by using historical data from approved drugs to validate the predictive ability of brain organoid models, comparing brain organoid data with animal models and human patient data, and establishing dose–response relationships across models. Next, achieving precise drug screening is also significant, such as using brain organoid models to evaluate the inhibitory effects of compounds on viral replication and neuronal damage, and screening candidate drugs for *in vivo* trials. Finally, it is necessary to establish a dynamic feedback loop, which integrates clinical data and patient sample feedback from clinical trials into organoid models to optimize model predictive ability. The “organoid–clinical–organoid” closed loop can then continuously correct the drug development path.

### Neurotoxicity assessment

Additional applications of brain organoids in drug research include toxicity and drug safety assessment. Drug-induced neurotoxicity, including structural and functional neurotoxicity, is a significant reason for the discontinuation of candidate drug development (Lieberthal et al., 2024). Therefore, early identification of the neurotoxicity risks of candidate drugs is crucial. 3D brain organoids/models have been employed to investigate the effect of marketed drugs and environmental toxins on human neuronal development. For instance, Arzua et al. (2020) used human brain organoids to model alcohol-induced neurotoxicity, providing insights into the mechanisms of fetal alcohol spectrum disorders. They exposed brain organoids to different ethanol concentrations and found that alcohol-exposed organoid neurons exhibited substructural changes, such as mitochondrial cristae disruption, reduced mitochondrial matrix density, cytoskeletal disorder, and mitochondrial dysfunction with metabolic stress. Another study used brain organoids to evaluate the developmental neurotoxicity of paroxetine, a selective serotonin reuptake inhibitor commonly used to treat depression during pregnancy. The results showed that paroxetine caused an 80% reduction in synaptic marker expression, a 60% decrease in neurite growth, and a 40%–75% reduction in the overall oligodendrocyte population at therapeutic blood concentrations (Zhong et al., 2020). This suggests that therapeutic concentrations of paroxetine induce abnormal brain cell development, resulting in adverse effects.

Environmental factors can potentially cause nerve damage. Wang and Matsushita (2021) examined the effects of excessive exposure to heavy metals, including cadmium, lead, and mercury, finding that these metals can induce neurotoxic effects by disrupting adult neurogenesis. Pamies et al. (2018) used a brain organoid model to investigate the effects of acute exposure to rotenone (a natural insecticide and complex I inhibitor), finding that the toxicity of rotenone varied depending on the differentiation state of neurons, which exhibit higher reactive oxygen species and more severe mitochondrial dysfunction at early stages.

These studies highlight the great potential of brain organoids for drug screening and toxicity assessment. However, like all model systems, several limitations and challenges remain to be addressed. First, current human organoids exhibit heterogeneity and an inevitable batch effect. To address this problem, simplifying and standardizing the production protocols is essential for facilitating large-scale production. Additionally, while brain organoids replicate pathophysiological states more accurately, they may lack inter-lineage signaling among neural, ECs, glia, and immune cells, reducing their fidelity to native organs. Although some researchers have proposed methods for assembling vascularized brain organoids with glial cells, the high complexity of assembly is difficult to balance with the cost of large-scale brain organoid production. Finally, at the systemic level, a significant flaw in most organoid drug screening models is that they excessively focus on the effects of drugs on brain organoids while ignoring the impact of drugs on other organs. Because of this flaw, future preclinical drug screening may involve a combination of multiple screening methods, such as the use of brain organoids for the initial screening of hundreds of candidate compounds, followed by validation in animal models.

## Applications of Brain Organoids in Nervous Disorder Modeling

### Neurodevelopmental disorders

**Figure 4:**
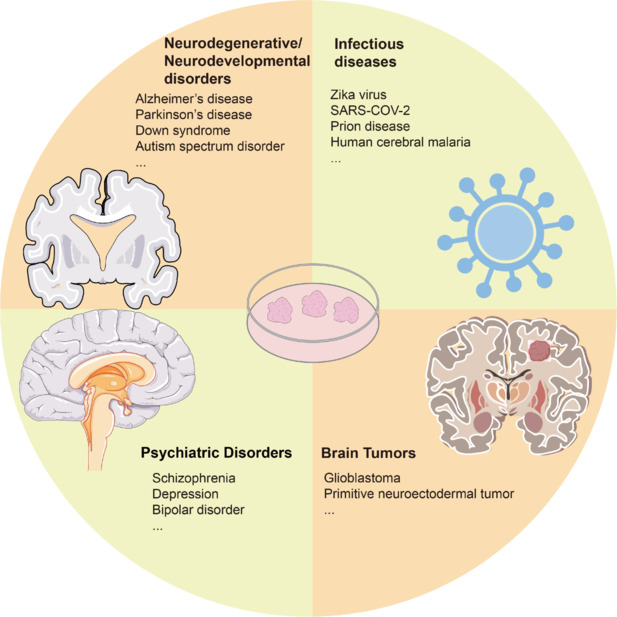
Application of brain organoids in neurological disorder modeling.

Research on most CNS disorders has reached a bottleneck because of the limitations of existing models. In contrast, the emergence of brain organoid models presents new opportunities for mechanistic research and the development of novel therapeutic strategies (**Figure 4**).

#### Autism spectrum disorder

Autism spectrum disorder (ASD) is a neurodevelopmental disorder with unclear etiology and genetic causes and is characterized by impairments in social communication, restricted interests, repetitive behaviors, and diverse clinical presentations of varying severity (Hirota and King, 2023). 2D cell culture systems have been used to study the cellular and molecular mechanisms of ASD. Research has shown that astrocytes derived from ASD individuals negatively modulate neuronal morphology and synaptogenesis by producing reactive oxygen species and interleukin-6, potentially offering a novel treatment approach for ASD (Russo et al., 2018). ASD-derived neurons exhibit impaired neurogenesis and reduced synaptogenesis, which can be reversed by insulin growth factor-1 (Marchetto et al., 2017). In another study, researchers co-cultured Ptenm3m4-mutant primary oligodendrocytes with wild-type dorsal root ganglion neurons and found an inability to properly myelinate axons, explaining the white matter abnormalities in ASD (Lee et al., 2019).

The emergence of brain organoid models offers new opportunities to explore the pathological mechanism of ASD. A study published in 2023 utilized human cortical organoids and revealed that the synaptic protein SYNGAP1 is expressed in radial glial cells and regulates cytoskeletal remodeling, differentiation timing, and cortical neurogenesis during the early development stage of ASD (Birtele et al., 2023). This finding offers a novel framework for understanding ASD pathophysiology. ASD-derived organoids exhibit accelerated cell cycles and overproduction of GABAergic inhibitory neurons. Overexpression of the transcription factor FOXG1 drives the overproduction of GABAergic neurons, and correcting FOXG1 dysregulation can restore the imbalance in GABAergic neuron fate (Mariani et al., 2015). CHD8 haploinsufficiency alters neurodevelopmental pathways, including accelerating inhibitory neuron development and slowing excitatory neuron development (Villa et al., 2022). By integrating human cortical organoid models with single-cell RNA-seq, researchers identified cell type developmental abnormalities resulting from haploinsufficiency of the ASD risk genes *SUV420H1*, *ARID1B*, and *CHD8* in multiple cell lines from diverse donors. The results revealed that cortical organoids with haploinsufficiency of the three ASD risk genes displayed asynchronous development of GABAergic neurons and deep-layer excitatory projection neurons, which resulted in aberrant circuit activity through distinct molecular pathways (Paulsen et al., 2022). Additionally, Li and colleagues developed a CRISPR-human organoid single-cell RNA-seq system by combining CRISPR, human organoid, and single-cell RNA-seq to characterize loss-of-function phenotypes of high-risk ASD genes across different cell types during early brain development in brain organoids. The results revealed that dorsal intermediate progenitor cells, ventral progenitor cells, and upper-layer (layers 2–3) excitatory neurons are the most vulnerable cell types (Paulsen et al., 2022).

#### Down syndrome

DS is another severe neurodevelopmental disorder. It is also known as trisomy 21, which results from an additional copy of chromosome 21, as the name suggests (Lana-Elola et al., 2024). To date, brain organoids have been widely used in the study of the mechanism of AD. Xu and colleagues created DS human brain organoids and observed an overproduction of OLIG2^+^ neural progenitor cells, resulting in excessive GABAergic interneuron generation. This anomaly was reversed by OLIG2 knockdown (Xu et al., 2019a). Another study focused on changes in microglia during DS brain development and revealed that DS microglia exhibit enhanced synaptic pruning, which is regulated by type I interferon signaling. Knocking out the type I interferon receptor IFNAR, which is encoded on chromosome 21, rescued the DS microglial phenotype (Jin et al., 2022). Jin et al. (2022) employed iPSC-derived brain organoids and chimeric mouse models to develop brain organoids containing glial cells. In this brain organoid model, changes in microglia during DS brain development were investigated, revealing that microglia display increased synaptic pruning, which is controlled by type I interferon signaling. Deleting the type I interferon receptor IFNAR on chromosome 21 restored the DS microglial phenotype. Another study based on brain organoid models demonstrated that DS brain organoids have diminished expression of layer II and IV cortical neuron markers and reduced proliferation, with increased DSCAM/PAK1 pathway activity. Downregulating or inhibiting this pathway reversed abnormal neurogenesis in DS organoids (Tang et al., 2021). Furthermore, inhibition of the DSCAM/PAK1 pathway corrected perinuclear mitochondrial clustering in cells from DS patients (Xu et al., 2022). Sali et al. (2022) have shown that individuals with DS are more susceptible to SARS-CoV-2-induced neuropathology. Choroid plexus-like epithelial cells are crucial in establishing SARS-CoV-2 infection in the brain. Shaker et al. (2024) developed brain organoid assembloids incorporating choroid plexus-like epithelial cells and reported that the TMPRSS2 protease present in these cells significantly contributes to the increased susceptibility of DS patients to SARS-CoV-2. In summary, 3D cortical organoids in vitro are valuable models for DS, which provides new avenues for understanding DS pathophysiology.

#### Microcephaly

Microcephaly is a neurodevelopmental disorder characterized by a head circumference more than two standard deviations below the age-matched mean and is associated with neural progenitor cell disruption (Woods, 2004; Huang et al., 2024b). Mutations in the ASPM gene, related to abnormal spindle-like microcephaly, are a common cause of recessive microcephaly, but ASPM knockout models do not fully mimic the phenotypes observed in human patients (von Wrede et al., 2022; Wu et al., 2022). In 2013, Lancaster et al. demonstrated premature neuronal differentiation in patient-derived iPSC organoids, indicating that organoids can recapitulate developmental disorders that are difficult to model in mice, including microcephaly. Qian et al. (2016) developed a small-scale biological platform to generate forebrain-specific organoids. When exposed to ZIKV, these forebrain organoids exhibited increased cell death, diminished proliferation, and reduced neuronal layer volume, resembling microcephaly, which provided initial evidence linking ZIKV to microcephaly. Studies based on brain organoids have revealed that abnormalities in several genes are linked to microcephaly. For example, cortical brain organoid models exhibit reduced proliferation of radial glial cells (RGCs) in microcephaly, and asparaginyl-tRNA synthetase 1 (NARS1) is essential for supporting RGC proliferation during human brain development. The critical role of NARS1 mutations in microcephaly was highlighted in brain organoids (Wang et al., 2020b). The AUTS2 gene has also been shown to play a significant role in the pathophysiological processes of microcephaly in brain organoids, including defects in neural progenitor cell proliferation (Fair et al., 2023). WDR62, a spindle pole-associated scaffold protein, is localized to the Golgi apparatus and moves to the spindle in a microtubule-dependent manner. By developing human brain organoid models, Dell’Amico and colleagues demonstrated that mislocalization or mutation of WDR62 results in severe neurodevelopmental defects (Dell’Amico et al., 2023). Pallavicini et al. (2024) used a brain organoid platform combined with CRISPR technology to construct brain organoids with biallelic variants of the citron rho-interacting serine/threonine kinase (CIT), including missense variants (CITKI/KI) and frameshift loss-of-function variants (CITFS/FS), to model microcephaly. The results revealed that both CITKI/KI and CITFS/FS organoids lost their cellular structural complexity, transitioning from pseudostratified to simple neuroepithelium, highlighting the utility of human forebrain organoid models in understanding the mechanisms underlying human microcephaly. In conclusion, brain organoid models offer a novel platform for investigating the pathophysiological mechanisms underlying microcephaly.

#### Timothy syndrome

Timothy syndrome (TS) is a severe neurodevelopmental disorder caused by mutations in L-type calcium channels and is characterized by autism, long QT syndrome, epilepsy, and other neuropsychiatric disorders (Chen et al., 2024). iPSC-derived cortical organoids from TS patients display notable functional and structural abnormalities, including heightened spontaneous excitatory postsynaptic currents and immature neuronal morphology (Revah et al., 2022). Birey et al. (2017) developed brain organoid assembloids that incorporated both the ventral and dorsal cortices, revealing aberrant migratory saltation in the interneurons of TS syndrome patients. Further research using similar forebrain organoid assemblies revealed that acute modulation of Cav1.2 can regulate the length but not the frequency of interneuron migratory saltations. This distinction arises because defects in saltation length are associated with abnormal actomyosin and myosin light chain phosphorylation. In contrast, defects in saltation frequency are linked to increased γ-aminobutyric acid (GABA) sensitivity (Birey et al., 2022). In conclusion, brain organoid models offer a valuable means to investigate the cellular mechanisms underlying TS.

In addition, brain organoids have been used to model other neurodevelopmental disorders, such as tuberous sclerosis complex (Mühlebner et al., 2016; Blair et al., 2018; Niu et al., 2024), Aicardi‒Goutieres syndrome (AGS) (Thomas et al., 2017), and Rett syndrome (RTT) (Hong et al., 2023). In summary, these studies have provided new perspectives for understanding the molecular basis of syndromic diseases, thereby offering new research targets for developing therapeutic strategies.

### Neurodegenerative disorders

Neurodegenerative disorders (NDDs) are a category of disease that significantly threatens the health of the elderly population and include AD, Parkinson’s disease (PD), Huntington’s disease (HD), amyotrophic lateral sclerosis (ALS), spinal muscular atrophy, Batten disease, and multiple sclerosis. Symptoms of NDD include memory decline, cognitive impairment, or dysfunction in other systems. The traditional models used to study NDD have certain limitations. The emergence of brain organoid models has partially overcome these limitations, aiding in a deeper understanding of the pathogenesis of NDD.

#### Alzheimer’s disease

AD is the most common neurodegenerative disease and is characterized neuropathologically by the deposition of amyloid-β (Aβ), which results in the formation of extracellular plaques, and hyperphosphorylated tau, which results in the formation of intracellular tangles (Hanseeuw et al., 2019). Zhao et al. used a large number of human iPSC-derived brain organoid models from healthy subjects and AD patients carrying apolipoprotein E (APOE) ε3/ε3 or ε4/ε4 genotypes to study AD-related phenotypes. The results demonstrated that APOE4 exacerbates neurodegenerative changes in iPSC-derived brain organoids from AD patients, but elevated Aβ levels in AD organoids are independent of the APOE genotype. More importantly, isogenic conversion of APOE4 to APOE3 attenuated AD-related phenotypes in iPSC-derived brain organoids (Zhao et al., 2020). In another study, researchers developed a novel method to create chimeric human brain organoids, revealing that the co-occurrence of APOE4 in astrocytes and neurons is necessary to increase phosphorylated tau levels in neurons (Huang et al., 2022a). A further study highlighted the effects of APOE4 on cell type-specific functions in AD pathology: APOE4 astrocytes presented reduced Aβ uptake and cholesterol accumulation, and APOE4 microglia-like cells presented morphological alterations that could hinder their ability to clear Aβ in AD and modify the inflammatory milieu of the brain (Lin et al., 2018). Disruption of the BBB is an additional risk factor for AD (Montagne et al., 2015). Consequently, Chen and colleagues exposed brain organoids to human serum to model the effects of BBB disruption in AD patients. Research has shown that serum-exposed brain organoids accurately replicate several AD-like pathologies, including elevated Aβ aggregates and phosphorylated tau levels, synaptic loss, and disrupted neural networks (Chen et al., 2021b). Additionally, dysregulation of 5-hydroxymethylcytosine (5hmC) is associated with AD, but how it contributes to AD pathology has not been fully explored. Compared with those in healthy organoids, studies have shown reduced levels of 5-hydroxymethylcytosine (5hmC) in forebrain organoids with PSEN1 and APP mutations from early-onset AD patients, alongside the upregulation of AD-related genes (Kuehner et al., 2021).

Brain organoids also play a significant role in investigating the therapeutic effects of various drugs on AD and in comparing their efficacy. In a study investigating the therapeutic effects of semaglutide on AD, researchers used organoid models and reported that semaglutide promoted reduced levels of Aβ, phosphorylated tau, and GFAP while increasing OXT levels (Zhang et al., 2024b). Brain organoid models also provide opportunities to explore the impact of environmental pollutants or neurotoxic organic chemicals on AD pathological processes. Researchers exposed brain organoids to varying concentrations of perfluoroalkyl and polyfluoroalkyl substances to investigate their toxicity, and the results revealed that the treated organoids exhibited Aβ accumulation and tau phosphorylation (Lu et al., 2024). In summary, brain organoid models have the potential to study various pathogenic processes of AD, including inflammation, lipid metabolism, genetic factors, and BBB disruption.

#### Parkinson’s disease

PD is a chronic neurodegenerative disorder characterized by the degeneration of midbrain dopaminergic neurons and the deposition of α-synuclein aggregates (Morris et al., 2024). Currently, there are multiple methods for constructing PD organoids. In 2016, researchers developed 3D midbrain-like organoids and detected functionally mature dopaminergic neurons and dopamine production within them. Additionally, neuromelanin-like granules produced by these organoids are structurally similar to those isolated from the human substantia nigra (Jo et al., 2016). Kim et al. (2019) generated midbrain organoids with leucine-rich repeat kinase 2 (LRRK2) mutations, characterized by reduced dopaminergic neuron expression and increased abnormal co-localization of α-synuclein and thioredoxin-interacting protein (TXNIP). Boussaad et al. (2020) used organoid models to confirm that PARK7 mutations cause PD and revealed that these mutations disrupt the binding motif of the small nuclear RNA U1. Rosety et al. (2023) used midbrain organoids derived from the iPSCs of patients carrying the heterozygous N370S mutation in the GBA gene to investigate the early developmental contributions of GBA mutations in PD. The results revealed that patient-specific midbrain organoids presented reduced numbers and complexity of dopaminergic neurons, increased neural progenitor cells, and oxidative stress-induced damage accompanied by premature cellular senescence. A separate study revealed that midbrain organoids generated from PD patient iPSCs exhibited increased apoptosis of tyrosine hydroxylase-positive (TH^+^) neurons and an imbalance in mitophagy and proliferation. Treatment with 2-hydroxypropyl-β-cyclodextrin resolved mitophagy issues and enhanced dopaminergic differentiation via various pathways (Jarazo et al., 2022). Additionally, PD organoids can be created by incorporating mutations in genes such as SNCA, GBA1, and DNAJC6 (Jo et al., 2021; Wulansari et al., 2021; Rosety et al., 2023). By combining organoid technology with genetic engineering, researchers have constructed various PD organoids and identified multiple genes and signaling pathways closely related to PD, significantly advancing PD research. In addition to serving as models for studying disease mechanisms, brain organoids have also played a significant role as validation models. In PD, it remains unclear whether cell types other than dopaminergic neurons in the substantia nigra are vulnerable. Wang et al. (2024f) used single-nucleus transcriptomic analysis to identify a unique neuronal cluster marked by the expression of RIT2, a PD risk gene, highlighting vulnerabilities in PD and then validated this finding using organoid models. In summary, brain organoids are indispensable for exploring the complexity of the molecular and cellular changes related to PD pathogenesis.

#### Huntington’s disease

HD is the most common autosomal dominant inherited neurodegenerative disorder and is caused by the expansion of cytosine-adenine-guanine (CAG) trinucleotide repeats in exon 1 of the huntingtin gene (HTT) (Mätlik et al., 2024). Current potential treatments for HD include the targeting of Huntington DNA and RNA, the clearing of Huntington protein, DNA repair pathways, and other strategies that target inflammation and cell replacement. However, no disease-modifying drugs have been approved for HD treatment (Tabrizi et al., 2022). Organoids hold significant promise for modeling HD and investigating its disease mechanisms. For example, Conforti et al. (2018) used HD patient-derived iPSCs and brain organoids to identify neurodevelopmental phenotypes associated with repeat expansion mutations. Transcriptomic analysis revealed that compared with healthy organoids, HD organoids have an immature transcriptional profile and disrupted cortical cell structure. An analysis of key developmental stages revealed that HD organoids remain in an early developmental phase and cannot fully differentiate or mature. These findings suggest a potential link between abnormal changes in early neurodevelopment and the eventual onset of neurodegenerative disease (Conforti et al., 2018). Studies have suggested that abnormal protein localization may be one of the pathogenic mechanisms of HD. In 2022, Liu and colleagues reported that heat shock factor 1 (HSF1) is overexpressed in the mitochondria of HD cell models, YAC128 mouse models, and human striatal organoids generated from HD-induced iPSCs. Inhibiting the mitochondrial localization of HSF1 ameliorates HSF1-induced mitochondrial abnormalities and improves HD-like pathology in animal models and human striatal organoids. These findings demonstrate that the abnormal localization of HSF1 may be one of the pathogenic mechanisms of HD and provides a promising target for HD treatment.

Furthermore, the striatal-nigral circuit is critical for regulating motor behavior, and its dysfunction may lead to a range of motor disorders associated with neurodegeneration, such as HD (DeLong and Wichmann, 2007). However, research on this circuit has been hindered by limitations in available models. Wu S et al. used iPSCs derived from HD patients to create human striatal-like organoids and midbrain substantia nigra-like organoids. By fusing striatal organoids with substantia nigra (SN) organoids, they constructed a striatal–nigral assembloid that partially recapitulates the features of the *in vivo* Str–SN circuit, including reciprocal projections and synaptic connections between GABAergic and dopaminergic neurons. Importantly, HD assembloids derived from patient iPSCs exhibit defective neuronal projections and reduced calcium signaling, demonstrating that this multiregional brain organoid platform can be used to study striatal–nigral defects effectively in HD (Wu et al., 2024b). In summary, HD organoids provide new resources for investigating HD mechanisms and exploring therapeutic strategies for HD.

### Psychiatric disorders

Psychiatric disorders are characterized by behavioral phenotype disturbances, multidimensional impairments in sensory integration, motor coordination, cognitive processing and affective modulation. The neural mechanisms involve abnormal connectivity and functional dysregulation across multiple brain regions, including the cortical-limbic-basal ganglia circuits. While rodent models serve as irreplaceable models for neuroscience, their limitations are particularly pronounced in psychiatric disorders. Evolutionary divergence spanning 65–96 million years has resulted in significant interspecies variations in CNS architecture among primates, particularly in neuronal subpopulations, synaptic networks, and glial functionality (Mestas and Hughes, 2004). Furthermore, disorder-specific symptom clusters (e.g., hallucinations, delusions, and affective blunting) are inextricably linked to phylogenetically advanced social cognition that is absent in rodents. Most importantly, primate and rodent cortices exhibit fundamental divergences in neurodevelopmental processes. Rodent neocortical neurons primarily originate from outer radial glia (oRGs) in the subventricular zone. In contrast, primates possess a unique outer subventricular zone characterized by the amplified mitotic activity of oRG, enabling massive expansion and migration of neural progenitor cells (Kennedy et al., 2021). These neuroanatomical distinctions have important implications for the study of neurodevelopmental disorders. Notably, hPSC-based brain organoids have successfully been used to model outer subventricular zone-like proliferative zones with characteristic expression profiles (e.g., GFAP, LIFR, and HOPX) (Walsh et al., 2024; Li et al., 2017b). This makes brain organoids excellent *in vitro* models of psychiatric disorders.

#### Schizophrenia

Schizophrenia is a complex psychiatric disorder with an incidence of approximately 287 cases per 100,000 people globally (Lv and Luo, 2025). Owing to the complexity of its pathophysiology, schizophrenia poses many challenges in treatment. Kathuria et al. performed transcriptomic analysis on brain organoids derived from patient-derived iPSCs and reported the dysregulation of genes involved in mitochondrial function and the regulation of excitatory and inhibitory pathways. Microelectrode array studies revealed no difference in baseline electrical activity in schizophrenia but revealed a weakened response to stimulation and depolarization, indicating neuronal dysfunction in schizophrenia (Kathuria et al., 2020b). In another study, researchers used brain organoids derived from patient iPSCs to model the neuropathology of schizophrenia. They reported altered progenitor cell survival and disrupted neurogenesis in these schizophrenia organoids. Further single-cell sequencing revealed that the main difference in schizophrenia organoids lies in the total number of diseases and neurodevelopmental factors at the molecular level. The transcription factor BRN2 and growth factor PTN were identified as mechanistic substrates for neurogenesis and cell survival in schizophrenic organoids (Notaras et al., 2022). Similarly, organoid models of schizophrenia present a greater percentage of ECs, with significant enrichment of genes related to angiogenesis, vascular regulation, and inflammatory responses in ECs (Stankovic et al., 2024). Previous clinical evidence suggests altered striatal function in schizophrenia patients, but research on the corresponding cellular and molecular mechanisms is limited. Sawada et al. generated ventral forebrain organoids from iPSCs derived from the dura mater fibroblasts of schizophrenia patients. Single-cell RNA sequencing analysis revealed differences in developmental trajectories between ventral forebrain organoids from schizophrenia patients and normal controls. Notably, inhibitory neurons in schizophrenia patients exhibit accelerated maturation. Genes upregulated in inhibitory neurons of schizophrenia ventral forebrain organoids significantly overlapped with those upregulated in postmortem caudate tissues of schizophrenia individuals, demonstrating that these ventral forebrain organoids effectively model disease-related cell type-specific neurodevelopmental phenotypes (Sawada et al., 2024).

#### Major depression disorder

Major depressive disorder (MDD) is one of the most common mental health conditions, yet its pathophysiology remains incompletely understood. Although the serotonin deficit hypothesis of depression has faced increasing scrutiny, it remains a cornerstone theoretical framework for MDD pathogenesis (Page et al., 2024). As a key monoamine neurotransmitter, serotonin (5-hydroxytryptamine, 5-HT) modulates diverse physiological processes, including affective states, sleep‒wake cycles and feeding behavior. This hypothesis posits that deficient synaptic 5-HT levels or aberrant signaling may underlie depressive symptomatology. Emerging research employing cerebral organoid models of MDD has provided further support for this hypothesis. For example, Lu et al. differentiated iPSCs from MDD patients into GABAergic interneurons and ventral forebrain organoids and reported that GABAergic interneurons presented altered neuronal morphology, abnormal electrophysiological hyperexcitability, and decreased calcium signaling. Transcriptomic analysis suggested that reduced expression of serotonin receptor 2C (HTR2C) may be responsible for deficits in neuronal activity. Furthermore, both HTR2C overexpression and pharmacological activation of HT2CR using Trazodone hydrochloride were demonstrated to ameliorate the observed impairments in neuronal activity (Lu et al., 2023). A separate investigation employing brain organoid models demonstrated that serotonin receptor 6 (5-HT6R) activation enhances neural progenitor proliferation and modulates human-specific neurogenic processes. Conversely, dysfunction or inhibition of 5-HT6R leads to premature differentiation of neural stem cells. Subsequent investigations revealed that 5-HT6R knockout mice exhibited depressive-like behaviors and impaired hippocampal neurogenesis. Using brain organoid and mouse models, the results suggest that modulation of 5-HT6R could be a potential therapeutic approach for alleviating depression (Wang et al., 2021d).

#### Bipolar disorder

Bipolar disorder, a psychiatric condition, also encounters considerable subjectivity in diagnosis and treatment because of its complex symptoms. Brain organoids generated from the iPSCs of bipolar disorder patients presented upregulated expression of immune signaling-related genes and downregulated expression of genes involved in cell adhesion, neurodevelopment, and synaptic regulation (Kathuria et al., 2020a). A separate study outlined a brain organoid model for bipolar disorder, revealing changes in neurodevelopment, increased neural network activity, and significant enrichment of gene targets of the transcriptional repressor REST, all of which were mitigated by lithium treatment (Meyer et al., 2024). Phalnikar et al. (2024) reported that bipolar disorder organoids presented neurodevelopmental abnormalities, such as proliferation and migration defects. Omics analysis revealed that the key genes LHX2, EMX2, PAX6, and HES1 are potentially crucial in this process. Compared with schizophrenia organoids, bipolar disorder organoids presented significant enrichment of genes related to calcium binding, neurotransmitter transport, ion binding, and transport regulation (Kathuria et al., 2023). Brain organoid models have been employed to explore the therapeutic effects and mechanisms of drugs for bipolar disorder. Osete et al. (2023) utilized brain organoids to investigate the therapeutic effects and mechanisms of lithium in bipolar disorder. These findings indicate that lithium treatment restores neuronal excitability in bipolar disorder, regulates pro-inflammatory cytokine secretion, and enhances the mitochondrial reserve capacity. In summary, organoids provide a more suitable *in vitro* model for studying the mechanisms and treatment strategies of mental illnesses.

### Brain tumors

Owing to the complex cell types and structures in the human brain, brain tumors are considered one of the most destructive types of tumors. To better understand the pathogenesis and treatment of brain tumors, various animal models have been established to simulate the pathological phenotypes and mechanisms of brain tumors (Jacques et al., 2010). However, owing to species differences, these mouse models cannot fully replicate the phenotypes of brain tumors. The advent of organoids has led to further progress in brain tumor research. Research published in 2018 introduced oncogenic mutations into brain organoids using transposons and CRISPR–Cas9-mediated mutagenesis to establish tumorigenic brain organoids. Screening revealed a combination of mutations leading to glioblastoma-like and CNS primitive neuroectodermal tumor-like tumors (Bian et al., 2018). In another study, researchers used CRISPR/Cas9 technology to integrate the HRas G12V-IRES-tdTomato construct into the TP53 locus via homologous recombination, creating a glioma cancer model. The tumor cells in this model exhibited an invasive phenotype and displayed a gene expression profile consistent with the mesenchymal subtype of human glioblastoma (Ogawa et al., 2018). Linkous et al. (2019) used patient-derived glioma stem cells and hESC-derived brain organoids to construct a glioma model mimicking glioblastoma. The model revealed that glioma stem cells migrated toward human brain organoids, invaded, and proliferated within the host tissue, forming tumors associated with glioblastoma phenotypes. Wang et al. (2024a) used glioblastoma-like organoids combined with single-cell transcriptomic analysis to reveal that NF1 mutations drive mesenchymal characteristics and identified several targets and drugs, such as DNA methylation and ECM degradation. In summary, these models have been instrumental in drug testing and pathogenesis research, providing new avenues for understanding the mechanisms of brain tumors and developing targeted treatment strategies (Xu et al., 2023a; Majc et al., 2024; Mangena et al., 2025).

### Infectious diseases

The CNS is generally resistant to pathogens because of the protective effect of the BBB. However, when the body’s immunity is weakened, a variety of pathogens can take the opportunity to invade the brain. Furthermore, many pathogens, such as SARS-CoV-2, ZIKV, and human immunodeficiency virus, show neurotropic properties and cause severe diseases of the CNS (Annadurai and Kanmogne, 2024; Shan et al., 2024; Wang et al., 2024e). However, owing to the complexity and vulnerability of the brain, the treatment of patients with CNS infection is challenging, which leads to poor prognosis and places a substantial economic burden on society (Srivastava et al., 2024; Wongsawat et al., 2024).

Owing to technical and ethical limitations, direct studies of the effects of infectious diseases on the human CNS are challenging. Many studies of infectious diseases rely on postmortem samples, immortalized cell lines, and animal models. However, these models do not fully represent the complexity of the human brain. Thus, brain organoids represent the most promising model and can provide a powerful in vitro platform for mechanistic research and the identification of novel therapeutic interventions.

#### Zika virus

ZIKV is the most studied virus in brain organoid models. This is due to the sudden and significant outbreak of ZIKV, which has promoted urgent research worldwide. Moreover, the lack of suitable animal models encouraged the development of brain organoid research. Several studies have reported that ZIKV infection of brain organoids results in a reduction in organoid size and surface folding (Li et al., 2017a, 2022b; Karvas et al., 2022; Schöbel et al., 2024). Most studies have consistently shown that ZIKV mainly infects early cortical neurons of brain organoids and promotes the premature differentiation of neural progenitor cells (Xu et al., 2019b; Cavalcante et al., 2020), whereas infection at later stages can lead to susceptibility of astrocytes (Janssens et al., 2019; Rubio-Hernández et al., 2023). These studies collectively indicate that ZIKV infection of brain organoids leads to destruction of the brain cell organization structure; increased neuronal death; decreased neural progenitor cell proliferation; and increased susceptibility of the ventricular, subventricular, and intermediate zones (Salick et al., 2017; Vancamp et al., 2021).

#### SARS-CoV-2

COVID-19 is an acute respiratory syndrome caused by SARS-CoV-2. SARS-CoV-2 is an enveloped single-stranded RNA virus that belongs to the Coronaviridae family. Studies have reported that patients with SARS-CoV-2 infection usually suffer from CNS disorders, such as headache, seizures, loss of smell and taste, confusion, neuroinflammation, and psychiatric disorders (Tyagi et al., 2023; Cheyne et al., 2024). SARS-CoV-2 RNA and protein were detected in cerebrospinal fluid samples obtained from patients with SARS-CoV-2 infection, suggesting that SARS-CoV-2 has neurotropic properties. Additionally, angiotensin converting enzyme 2 (ACE2), the target receptor of SARS-CoV-2, widely exists in brain cells, including neurons, astrocytes, oligodendrocytes and ECs, which also suggests that SARS-CoV-2 may directly infect brain cells (Jackson et al., 2022; Wang et al., 2024e). To date, numerous studies have used brain organoids to investigate the neurotropic, neurodestructive effects and therapeutic strategies for SARS-CoV-2. Using iPSC-derived brain organoids to examine SARS-CoV-2 neurotropism, Pellegrini et al. reported that the viral receptor ACE2 was expressed in mature lipoprotein-rich choroid plexus cells. These findings prove that SARS-CoV-2 infection damages the choroid plexus epithelium, leading to pathogens, immune cells, and cytokines entering the cerebrospinal fluid and brain. Interestingly, SARS-CoV-2 rarely infects neurons (Pellegrini et al., 2020). The same conclusion was also confirmed in a mouse model (Qiao et al., 2024). Further studies established brain organoids derived from DS patient iPSCs, which consisted of cortical neurons and ChP-like epithelium (ChPCO). These assembloids recapitulate abnormal DS cortical and ChP-like epithelial ciliogenesis. By establishing SARS-CoV-2 infection, researchers reported that ChPCO promoted SARS-CoV-2 infection and replication in cortical neurons, whereas inhibiting TMPRSS2 and furin activity reduced viral replication in DS-ChPCOs (Shaker et al., 2024). These findings shed light on the mechanism of SARS-CoV-2-induced DS pathology. To explore the effects of SARS-CoV-2 at the genetic level, scientists have performed single-cell or bulk RNA sequencing of SARS-CoV-2-infected human brain organoids. Single-cell RNA analysis of these infected human brain organoids further confirmed the widespread infection response of the virus in NPCs, radial glia, and neurons, leading to nerve damage (Song et al., 2021; Hu et al., 2024).

In addition, brain organoids provide new insights into various infectious diseases/viruses, such as HSV1 (Rybak-Wolf et al., 2023), human cytomegalovirus (Ijezie et al., 2023), prion disease (Groveman et al., 2021), and human cerebral malaria (Harbuzariu et al., 2022). However, the brain organoid model of infectious diseases still has several limitations. First, both immature and mature brain organoids exhibit gene expression patterns similar to those of the fetal human brain. This is an advantage when studying pathogens that cause congenital neurological disorders during fetal neurodevelopment. However, applying these findings to adult brain infections is less evident than extrapolating the results to human viral brain infections in infants or children. Second, brain organoids generally do not contain microglia, which are crucial for brain development, pathogen spread, immune response activation, and pathogen-mediated brain pathology. By incorporating immune cells into co-cultures, brain organoid assembloids containing microglia have been generated (Park et al., 2023; Kong et al., 2024; Mrza et al., 2024), which will advance the study of the role of microglia in the mechanism of viral infection. Another significant limitation is the lack of a BBB. This hinders research on pathogen neural invasion because the lack of a BBB makes it impossible to determine whether pathogen-induced neurological complications are caused by direct infection in the CNS or by an induced immune response. Therefore, the development of brain organoids combined with the BBB model may play an essential role in the research of brain infectious diseases.

## Therapeutic Potential of Brain Organoids Transplantation

### Advantages of brain organoids transplantation therapy

Due to organoids exhibiting higher tissue complexity than 2D cell cultures, they allow researchers to simulate tissue development and disease more accurately. Brain organoids derived from iPSCs are generated by reprogramming somatic cells from human subjects. This process suggests that organoids preserve the subjects’ genetic background, providing substantial potential for personalized medicine (Wu et al., 2023a). The viability of transplanting multiple organoids—including those containing CNS structures—into hosts has been demonstrated in animal models (Takebe et al., 2013; Nie et al., 2018; Tsuchida et al., 2020; Watanabe et al., 2022). For example, various types of organoids have been used for liver transplantation (Tadokoro et al., 2024), intestinal transplantation (Zhang et al., 2023; Tadokoro et al., 2024), repairing spinal cord lesions (Xu et al., 2023b), and as alternative therapies for retinal degeneration (Watari et al., 2023).

Brain organoids exhibit greater complexity than other specialized organoids. Recovery from neural injury entails a series of neurogenic processes, including vascularization (Liu et al., 2023b), neural differentiation (Liu et al., 2024), synaptogenesis (Hosseini et al., 2022), and axonal growth. Given these requirements, brain organoids can be utilized for neural regenerative therapies in various diseases, owing to the unique differentiation potential of stem cells. Brain organoids consist of various cell types, including neural progenitor cells and differentiated cell types, developed *in vitro* (Tidball et al., 2023; Liang, 2024; Sabogal-Guaqueta et al., 2024). This cellular diversity aligns with the needs of regenerative medicine, which aims to reconstruct injured tissue into a functional whole rather than merely restoring individual cells. Transplanted brain organoids contain numerous neural progenitor cells that can migrate and differentiate into appropriate cells in response to signaling molecules from the surrounding environment. Differentiated neurons extend their axons within the transplanted organoids and adjacent host tissues, guided by growth factors and the extracellular matrix (ECM) (Kitahara et al., 2020). This process of axonal growth facilitates the formation of synapses (synaptogenesis) between newly differentiated neurons, thereby reconnecting neural circuits between the injured area and the host tissue (Fan et al., 2024).

Stem cell-based neural therapies have facilitated neural regeneration, axonal growth, and synaptogenesis to some extent (Rahimi Darehbagh et al., 2024; Sun et al., 2024c). However, several limitations remain, such as the inability to induce vascularization and unstable long-term effects. In contrast, brain organoids are not merely isolated stem cells but complex structures composed of diverse neural and glial cells (Adeyeye et al., 2024; Lisowski et al., 2024). Furthermore, there have been reports of brain organoids with glial cells and those with vascular systems (Koh and Hagiwara, 2024; Lisowski et al., 2024; Wu et al., 2024a). These brain organoids integrate seamlessly into the transplant site, improving cell survival rates and providing a stable environment for neural therapy. Additionally, transplanted stem cells may fail to differentiate into specific types of neurons or other required cells, potentially leading to poor functional recovery, side effects, or even uncontrolled proliferation and tumor formation (Miller et al., 2023; Sauerer et al., 2023; Mumby et al., 2024). The cellular environment of brain organoids closely resembles the physiological CNS, and these structures can be exogenously regulated before transplantation, enabling the pre-adjustment of most brain organoid characteristics *in vitro* (Marton and Pașca, 2020; Sandoval et al., 2024). This enables most brain organoid characteristics to be pre-adjusted *in vitro*, ensuring tissue compatibility and the correct differentiation direction before transplantation. For instance, Daviaud et al. (2018) transplanted neural stem cells and brain organoids into the mouse cortex, respectively. Their results showed that brain organoid transplantation exhibited higher survival rates and robust vascularization from the host brain compared to dissociated neural progenitor cell transplantation. This demonstrates that CNS organoid transplantation is superior to neural stem cell transplantation. Researchers have confirmed that brain organoid grafts exhibit superior therapeutic outcomes, evidenced by higher survival rates and enhanced neural differentiation. The microenvironment, tissue compatibility, and cellular homogeneity of transplanted organoids are critical factors that enable their outstanding neural reconstruction capabilities.

### Application of brain organoid transplantation therapy in acute brain injury

Current research on brain organoid transplantation therapy primarily focuses on acute brain injury (ABI). Conditions such as ischemic stroke (IS) and traumatic brain injury (TBI) are categorized as ABI and are characterized by sudden damage to the brain parenchyma (Frisvold et al., 2023; Reyes-Esteves et al., 2023). Current treatments for ABI remain largely conservative with limited efficacy (Kowalski et al., 2021). While traditional animal models and *in vitro* cell culture systems have provided valuable mechanistic insights, they are insufficient to fully capture the pathophysiological complexity of ABI (Xu et al., 2017; Liu et al., 2023a). Therefore, additional experimental models are needed to replicate the key features of ABI more accurately.

#### Traumatic brain injury

To investigate the therapeutic potential of brain organoid transplantation for TBI, multiple studies have developed TBI models with different degrees of severity. Kim et al. (2022) constructed iPSC-derived cortical organoids and transplanted them into a mild TBI mouse model at 8 weeks post-injury. The results demonstrated that the transplanted brain organoids survived within the TBI mouse brain, suggesting successful reconstruction of cortical tissue. Fluoro-Jade B staining demonstrated a significant decrease in Fluoro-Jade B-positive cells, suggesting that the brain organoids exhibit neuroprotective properties. Vascular-like structures were observed in the brain organoids within 14 days post-transplantation. Immunostaining results also revealed that most transplanted organoid cells differentiate into immature neurons. Furthermore, novel object recognition testing was used to evaluate cognitive improvements in the injured mice, and the results revealed that the transplanted mice showed a stronger preference for new objects, indicative of cognitive improvement (Kim et al., 2022). Bao et al. (2021) transplanted hESC-derived brain organoids *in vitro* to assess the therapeutic effects on moderate to severe TBI. The transplanted brain organoids exhibited proper growth and differentiation over time *in vivo*, accompanied by decreased GFAP expression, suggesting that transplantation facilitated neural repair and enhanced glial scar formation. More importantly, the transplanted brain organoids also exhibited spontaneous action potentials under simulation, indicating their ability to transmit neural signals. Meanwhile, the Morris water maze test revealed improvements in the spatial learning and memory abilities of the mice. This indicates that brain organoids implanted at the lesion site differentiated into cortical neurons, formed long projections, and reversed spatial learning and memory deficits. This highlights a potential therapeutic approach to TBI.

To ensure the effectiveness of transplantation, cortical organoids can be appropriately vascularized (Sun et al., 2024b). Mansour et al. (2018) transplanted human brain organoids into the brains of immunodeficient mice, demonstrating that they successfully integrated with the host vascular system, formed synaptic connections and axonal projections with host neurons, and maintained their intrinsic neuronal activity. Daviaud et al. (2018) compared the survival rates and differentiation degree after the transplantation of brain organoids and dissociated neural progenitor cells. They found that transplanted brain organoids contained more neural stem cells than dissociated neural progenitor cells and exhibited rich vascularization on their surfaces. Additionally, brain organoids exhibited multi-lineage neural differentiation at 2–4 weeks post-transplantation, suggesting that the vascularization and cellular organization of brain organoid transplantation are more effective than those of neural stem cells.

In addition, the age of brain organoids may play a crucial role in influencing the therapeutic outcomes of TBI treatment. A study conducted by Kitahara et al. (2020) demonstrated that transplanting 6-week-old brain organoids into TBI mice resulted in graft overgrowth and more active axonal extension than the transplantation of 10-week-old brain organoids. Another study performed similar experiments with 55- and 85-day-old brain organoids, showing that 55-day-old organoid implantation exhibited superior therapeutic effects in terms of synaptic regeneration, anti-inflammation, and neurogenesis (Wang et al., 2020d). In conclusion, these studies highlight the significance of the age of brain organoid transplantation for the recovery of injury lesions.

#### Ischemic stroke

Cerebrovascular accidents or strokes are the leading causes of disability and death globally (Hilkens et al., 2024). The management of acute strokes typically includes the intravenous administration of recombinant tissue plasminogen activator and/or arterial recanalization through mechanical endovascular thrombectomy. However, despite optimizing medical risk factors, lifestyle modifications, and rehabilitation efforts, there are no widely applicable regenerative methods for repairing neuronal damage (Hwang et al., 2024).

Brain organoids are promising candidates for this therapy due to their extensive differentiation potential and capacity to replace tissue lost to hypoxia. Wang et al. (2020c) transplanted 55-day-old forebrain organoids into a rat middle cerebral artery occlusion stroke model. The results demonstrated that forebrain organoid transplantation significantly reduced infarct volume and improved neuromotor function. The transplanted forebrain organoids exhibited multi-lineage differentiation potential, mimicking *in vivo* cortical development, supporting region-specific reconstruction of the motor cortex, generating neurotransmitter-related neurons, and establishing synaptic connections with the host brain through in situ differentiation and cell replacement in stroke. One study explored the efficacy and underlying mechanisms of cerebral organoid transplantation in stroke (Wang et al., 2020c). Cao et al. (2023a) cultured human brain organoids derived from hPSCs and transplanted them into the border zone between the infarct core and peri-infarct region of stroke mice. The results demonstrated that, months later, the transplanted organoids had survived well in the infarct core, differentiated into target neurons, repaired infarcted tissue, sent axons to distant brain targets, and integrated into the host neural circuitry, restoring sensorimotor function in stroke mice. However, single-cell transplantation derived from organoids failed to repair the infarcted tissue (Cao et al., 2023a). Subsequently, Cao et al. (2023b) constructed human brain organoids resembling the MGE domain (human MGE organoids) from iPSCs and transplanted them into the infarcted cortex of stroke mice. The findings revealed that the transplanted human MGE organoids thrived—predominantly differentiated into GABAergic interneurons—and significantly improved sensorimotor deficits in stroke mice.

These studies propose novel strategies for reconstructing infarcted tissue through brain organoid transplantation and demonstrate superior efficacy compared to stem cell transplantation. Despite this progress, the methods for producing and analyzing brain organoids are not fully developed and require further preclinical research (**Additional Table 2**).

**Additional Table 2:** Recent preclinical studies on brain organoid transplantation

Author	Purpose	Type	Animal	Achievement
Kitahara et al., 2020	Repairing TBI	Brain organoid	Cynomolgus monkey, mouse	Transplanted organoids extended axons in the brains of cynomolgus monkeys and mice
Wang et al., 2020d	Repairing TBI	Brain organoid	Mouse	Transplantation of 55-day-old brain organoids enhanced neuromotor function and decreased cerebral injury
Dong et al., 2021	Repairing TBI	Sheared brain organoid	Mouse	The transplanted brain organoids survived, matured, and established subcortical projections
Kim et al., 2022	Repairing TBI	Brain organoid	Mouse	Transplantation of 8-week-old brain organoids reduced neuronal cell death, restored microvascular density, and promoted neurogenesis
Bao et al., 2021	Repairing TBI	Brain organoid	Mouse	Transplanted organoids differentiated into cortical neurons, formed long projections, and reversed spatial learning and memory deficits
Cao et al., 2023a	Repairing stroke	Brain organoid	Mouse	Brain organoids integrate into host neural circuits, thereby alleviating sensorimotor deficits in stroke mice
Cao et al., 2023b	Repairing stroke	Brain organoids resembling the MGE domain	Mouse	Transplanted organoids survive well and significantly restore sensorimotor deficits in stroke mice
Shi et al., 2020	Investigating adaptability of brain organoids	Vascularized cortical organoid	mouse	Vascularized cortical organoid transplantation reduces hypoxia and cell death, reconstructs the graft-host vascular system, and promotes neurodevelopment in organoids
Wilson et al., 2022	Investigating adaptability of brain organoids	Cortical organoid	Mouse	Transplanted organoids develop vascularization and, evoke electrophysiological responses that match the surrounding cortex
Jgamadze et al., 2023	Investigating adaptability of brain organoids	Forebrain organoid	Mouse	Transplanted forebrain organoids successfully integrate with the cortex and repair cortex circuitry
Schafer et al., 2023	Investigating adaptability of brain organoids	Vascularized brain organoid with microglia	Mouse	The transplanted organoids successfully integrate, and the microglia actively surveil environment, respond to damage, and react to systemic inflammation
Daviaud et al., 2018	Comparison of neural stem cells and brain organoid transplantation	Brain organoid	Mouse	Transplanted brain organoids exhibited higher survival rates and robust vascularization

TBI: Traumatic brain injury.

First, critical parameters, such as organoid age, size, and transplantation timing and regions significantly influence post-transplantation improvement, with optimal conditions varying according to disease type (Kitahara et al., 2020; Wang et al., 2020d; Dong et al., 2021; Kelley et al., 2024; Yamagami et al., 2024). For instance, the outcomes of neural progenitor cell transplantation are highly dependent on cellular quantity and temporal alignment with injury progression (Ji et al., 2024). Another major challenge is ensuring that transplanted organoids can successfully establish functional connections with host tissues. This involves not only physical integration but also the effective transmission of electrophysiological and chemical signals. Excitingly, preliminary studies have achieved structural and functional integration between transplanted brain organoids and host tissue (Revah et al., 2022; Jgamadze et al., 2023). For example, Jgamadze et al. (2023) transplanted human brain organoids derived from human iPSCs into the injured visual cortex of rats. The transplanted organoids remained active for three months and exhibited mature neuronal cell types. Notably, the viral tracing revealed connectivity between the transplanted organoids and the host’s visual circuitry, with retinal output signals transmitted to the organoids through synaptic connections. Furthermore, electrophysiological recordings of neuronal activity indicated that the transplanted organoids exhibited light responsiveness and local field potential activity, suggesting that neurons within the transplanted organoids are capable of specific responses to visual stimuli (Jgamadze et al., 2023).

**Figure 5:**
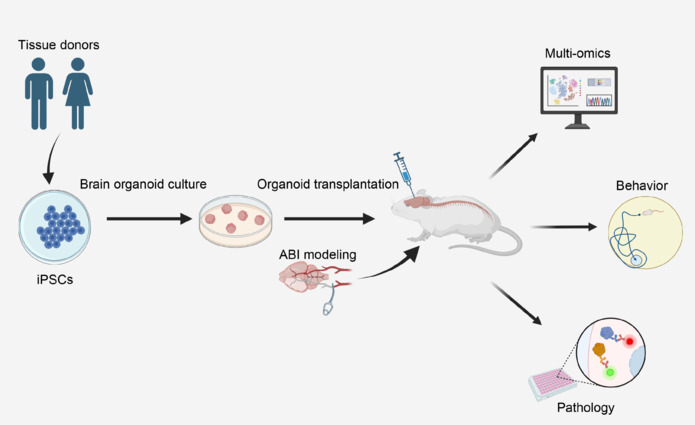
Simple transplantation procedure for ABI with brain organoids. ABI: Acute brain injury; iPSCs: human induced pluripotent stem cells.

However, translational barriers extend beyond biological variables. Immunogenicity and safety concerns remain unresolved, particularly regarding the use of human brain organoids in rodent models. Although it is generally believed that iPSC derivatives from autologous cells can avoid some immune rejection (Zhou et al., 2024), the mechanisms of redifferentiation and proliferation after brain organoid transplantation remain unclear, and the existence of transplant immunogenicity requires more empirical evidence. Notably, current *in vitro* culture processes for brain organoids contain many difficult-to-control components such as mouse embryonic fibroblasts, essential growth factors, and immunologically active substances, posing significant safety concerns for transplantation therapy (Chiaradia et al., 2023). Additionally, even with careful screening and processing, brain organoids may still contain incompletely differentiated stem cells with high proliferative capacity, increasing the risk of tumorigenesis post-transplantation, although systematic studies and clinical evidence are currently lacking. In summary, while brain organoid transplantation displays remarkable therapeutic potential, robust preclinical validation will be essential for advancing its clinical translation. The simple transplantation procedure is illustrated in **Figure 5**.

## Ethical Issues of Brain Organoid Research

Human brain organoids offer unique opportunities to explore the human brain, including its development and diseases. Researchers are utilizing these progressively sophisticated models to investigate disease mechanisms, assess the neurotoxicity or therapeutic efficacy of compounds, and generate patient-specific organoids (Miura et al., 2022). However, as brain organoids evolve in complexity, new concerns arise regarding the need for additional ethical and regulatory frameworks to guide brain organoid research (Boyd, 2024). The debates primarily center around the following aspects: challenges related to informed consent, clinical applications of organoids, and the consciousness and moral status of organoids (Koplin and Savulescu, 2019; Lavazza, 2020; Barnhart and Dierickx, 2022, 2023; Koplin, 2024).

### Challenges related to informed consent

The conventional guideline for reusing human tissue in research is “consent or anonymization” (Olatunji et al., 2024). Researchers should obtain donor consent or anonymize the samples. Complete anonymization of tissues is believed to safeguard donor interests and privacy sufficiently. However, in organoid research, anonymization and de-identification may undermine the scientific and clinical value of organoids. For instance, decoupling organoids from personal and biological data diminishes their medical value, as diagnostic, therapeutic, or other relevant findings cannot be returned to donors (Lensink et al., 2020). Furthermore, valuable data may be lost without long-term donor tracking (Lensink et al., 2021b). For donors, anonymization and de-identification result in losing control of their samples. However, tissue donors wish to control the research use of organoids derived from their tissues (Lensink et al., 2021a).

When anonymization is possible, appropriate informed consent is required. However, the broad potential clinical applications of organoids complicate the anticipation and description of future uses and storage of donor samples in informed consent documents (Haselager et al., 2020). For instance, although most brain organoids are currently limited to laboratory research, some donors may be concerned about potential future applications, including cloning, transplantation, and human enhancement (Boers et al., 2018; Lensink et al., 2021a). Regarding bodily integrity and identity, donors may feel a stronger connection with complex organoid models derived from their tissues than their cell lines, thus potentially requiring stricter informed consent procedures. For example, Bredenoord et al. (2017) demonstrated that organoids derived from donor tissues are perceived by donors as entities closely tied to their personal identity, suggesting that donors possess a nuanced understanding of the closeness between their bodies and organoids. Haselager et al. (2020) interviewed 28 patients and non-professionals about their views on the development of brain organoids, with respondents supporting the use of brain organoids under conditions of complete donor understanding and good governance but opposing potential misuse. These cases highlight the need for more comprehensive integration of informed consent procedures for brain organoid research.

### Ethical considerations in organoid clinical applications

Patient-derived organoids provide a promising platform for drug testing. Organoids that evaluate drug efficacy allow patients to avoid exposure to drugs with severe side effects (Park et al., 2021; Zhu et al., 2024). This makes organoids a novel form of evidence for clinicians and enables personalized medicine. However, ethical concerns must be addressed. First, maintaining a close link between patients and their organoids poses a risk of privacy breaches when organoids are used for personalized treatment (Lensink et al., 2020; Kataoka et al., 2025). Second, the substantial time and financial costs of generating organoids, combined with their donor-specific efficacy, create barriers to personalized therapy (Pode-Shakked and Devarajan, 2022; Carvalho et al., 2023). Third, although organoids closely mimic original organs under physiological conditions, a significant portion remains unrepresented (Smirnova et al., 2023). This is also a point of controversy in organoid technology. Fourth, the high specificity and cost of organoids make large-scale cohort testing impractical, resulting in potential undetected risks (Skardal et al., 2020). Finally, drug screening studies utilizing patient-derived organoids obscure the boundaries between basic research and clinical applications (Lensink et al., 2020, 2021a). Given the significant differences in ethical regulations between basic research and clinical practice, an appropriate regulatory framework is currently lacking.

### Brain organoids and consciousness

The debate surrounding theories of consciousness is long-standing and contentious, with some issues at the boundary between empirical science and philosophy, lacking consensus on necessary or sufficient criteria (Klincewicz et al., 2025). From basic awareness of stimuli to higher-order self-awareness, most definitions of consciousness require some form of perception, phenomenal experience, and information integration. Theories also diverge on the topic of specific mechanisms, such as higher-order theories of consciousness, global workspace theory, and integrated information theory (Brown et al., 2019; Seth and Bayne, 2022). Despite no consensus among neuroscientists on defining or quantifying consciousness, advances in sophisticated multi-region brain organoids have sparked debates about their potential for consciousness. Some studies suggest that complex brain organoids may have the potential to develop consciousness. For example, Quadrato et al. (2017) developed relatively mature brain organoids that survived for over 9 months and exhibited differentiation of photoreceptor-like cells with light-sensitive proteins. These photosensitive cells can respond to non-invasive light stimuli; some studies generated human cortical organoids and monitored their electrical activity, finding that 6-month-old organoids exhibited neural activity resembling mid-to-late-stage fetuses (Trujillo et al., 2019; Fitzgerald et al., 2024). Ghatak et al. (2021) established a physiological testing platform using electrophysiological patching, calcium imaging, and multi-electrode arrays to analyze familial AD-mutant brain organoids, revealing enhanced spontaneous action potentials, slow oscillations (**~**1 Hz), and hypersynchronous network activity.

Despite emerging studies, most experts in the field still regard brain organoids as non-sentient human tissues lacking independent moral status (Lovell-Badge et al., 2021; Hyun et al., 2022; Pașca et al., 2022; Koplin, 2024). This consensus is underpinned by several key arguments. First, while electrical activity in brain organoids may signify the formation of rudimentary neuronal circuits and responsiveness to external stimuli such as light, it does not equate to subjective experience or advanced information-processing capabilities. Such activity remains a far cry from the sophisticated neural dynamics of consciousness. Second, natural consciousness is widely understood to necessitate whole-brain integration, particularly involving systems like the thalamocortical network, as well as sensory input for perception, memory, and intentionality—features that current organoids conspicuously lack due to their limited structural complexity. Third, from a philosophical perspective, consciousness is often conceptualized as requiring dynamic interaction between an organism and its environment, as posited by the embodied cognition theory. In contrast, brain organoids, as isolated *in vitro* systems, are physically disengaged from environmental stimuli and bodily feedback, thereby precluding the possibility of subjective experience. Collectively, although brain organoids possess some brain-like structures, they currently lack the capacity to develop consciousness.

In the near future, brain organoids will become more complex. Due to the lack of communication capability in brain-like tissues, we cannot ascertain their developmental stage or capacity to generate conscious states. Therefore, developing tests to objectively evaluate consciousness capacity is crucial. Although no such tests have been designed explicitly for brain organoids, recent advances in assessing consciousness in non-communicative brain-injured patients at least provide similar frameworks. For instance, multi-electrode arrays or patch-clamp techniques could monitor neuronal firing patterns and synchronous oscillations (e.g., gamma waves and synchronized neural activity), where whole-brain-like high-frequency oscillations may indicate complex information processing (Manz et al., 2021; Zeng et al., 2024). Neuroimaging techniques such as calcium imaging can examine dynamic connectivity networks within organoids, analyzing whether they exhibit human brain-like network properties (Andersen et al., 2020; Chen et al., 2021a). A more sophisticated metric, the perturbational complexity index (PCI), is derived from integrated information theory. This method involves applying magnetic stimulation while recording neural responses using high-density EEG (Farisco and Changeux, 2023). PCI provides a numerical measure of the resulting neural responses’ complexity. A loss of integration reduces neuronal interactions, yielding low PCI values (spatially constrained responses), whereas a loss of differentiation results in uniform activity across regions, producing low PCI values (large but simple responses). A high PCI value requires both integrated and differentiated neural activity. Importantly, current methods only assess neural complexity and do not definitively prove consciousness. More groundbreaking theories and detection methods are required to explore whether consciousness exists in brain organoids.

## Limitations and Perspectives

Organoid technology is a powerful tool that has achieved many significant milestones in the field of neuroscience. However, several limitations and challenges remain. For example, although brain organoids containing microglia have been produced in basic research, there is currently no method for brain organoid assembly that includes key immune components, such as microglia, choroid plexus, and meninges, limiting the study of immune responses to viral exposure. The size of brain organoids remains limited, and their culture period typically spans several months, while lacking a timeline that mimics the normal development of the brain. This restricts long-term studies of neurodevelopmental trajectories. Additionally, although cortical organoids maintain a 3D tissue structure, they still lack the six-layer cellular structure. Finally, current brain organoids are unable to simulate complex multi-organ pathologies, such as the gut–brain (Góralczyk-Bińkowska et al., 2022; Wang et al., 2023), lung–brain (Granton et al., 2024; Xie et al., 2024), and liver–brain (Yin et al., 2022) axes. In terms of clinical applications, studies on brain organoid transplantation are still in the early stages. Clinical studies on brain organoid transplantation remain limited and necessitate comprehensive investigation.

## Conclusions

The development and application of 3D brain organoids represent a significant technological advancement with immense potential. They have the ability to model early neural development and diseases, providing valuable insights into neurological disorders. Furthermore, brain organoids hold significant value for drug screening, neurotoxicity assessment, and even transplantation therapies. Although there are still some challenges associated with this technology, with innovations in bioengineering and the maturation of organoid technologies, brain organoids will increasingly provide more accurate simulations of disease mechanisms, immune responses, and therapeutic interventions. This will broaden the application scope of brain organoids and ultimately enable patient-specific organoid transplantation therapies.

## Additional files:

***Additional Table 1:*** *Comparisons of the static and dynamic culture systems.*

***Additional Table 2:*** *Recent preclinical studies on brain organoid transplantation.*

## Data Availability

*All relevant data are within the paper and its Additional files*.

## References

[R1] Abreu CM, Gama L, Krasemann S, Chesnut M, Odwin-Dacosta S, Hogberg HT, Hartung T, Pamies D (2018). Microglia increase inflammatory responses in iPSC-derived human brainspheres. Front Microbiol.

[R2] Adeyeye A, Mirsadeghi S, Gutierrez M, Hsieh J (2024). Integrating adult neurogenesis and human brain organoid models to advance epilepsy and associated behavioral research. Epilepsy Behav.

[R3] Ahn Y, An JH, Yang HJ, Lee DG, Kim J, Koh H, Park YH, Song BS, Sim BW, Lee HJ, Lee JH, Kim SU (2021). Human blood vessel organoids penetrate human cerebral organoids and form a vessel-like system. Cells.

[R4] Almacellas Barbanoj A, Graham RT, Maffei B, Carpenter JC, Leite M, Hoke J, Hardjo F, Scott-Solache J, Chimonides C, Schorge S, Kullmann DM, Magloire V, Lignani G (2024). Anti-seizure gene therapy for focal cortical dysplasia. Brain.

[R5] Andersen J, Revah O, Miura Y, Thom N, Amin ND, Kelley KW, Singh M, Chen X, Thete MV, Walczak EM, Vogel H, Fan HC, Paşca SP (2020). Generation of functional human 3D cortico-motor assembloids. Cell.

[R6] Andrews MG, Kriegstein AR (2022). Challenges of organoid research. Annu Rev Neurosci.

[R7] Annadurai N, Kanmogne GD (2024). Structural and functional dysregulation of the brain endothelium in HIV infection and substance abuse. Cells.

[R8] Antón-Bolaños N, Faravelli I, Faits T, Andreadis S, Kastli R, Trattaro S, Adiconis X, Wei A, Sampath Kumar A, Di Bella DJ, Tegtmeyer M, Nehme R, Levin JZ, Regev A, Arlotta P (2024). Brain Chimeroids reveal individual susceptibility to neurotoxic triggers. Nature.

[R9] Ao Z, Song S, Tian C, Cai H, Li X, Miao Y, Wu Z, Krzesniak J, Ning B, Gu M, Lee LP, Guo F (2022). Understanding immune-driven brain aging by human brain organoid microphysiological analysis platform. Adv Sci (Weinh).

[R10] Aoki K, Higuchi T, Akieda Y, Matsubara K, Ohkawa Y, Ishitani T (2024). Mechano-gradients drive morphogen-noise correction to ensure robust patterning. Sci Adv.

[R11] Arzua T, Yan Y, Jiang C, Logan S, Allison RL, Wells C, Kumar SN, Schäfer R, Bai X (2020). Modeling alcohol-induced neurotoxicity using human induced pluripotent stem cell-derived three-dimensional cerebral organoids. Transl Psychiatry.

[R12] Ashina H, Eigenbrodt AK, Seifert T, Sinclair AJ, Scher AI, Schytz HW, Lee MJ, De Icco R, Finkel AG, Ashina M (2021). Post-traumatic headache attributed to traumatic brain injury: classification, clinical characteristics, and treatment. Lancet Neurol.

[R13] Babu HWS, Kumar SM, Kaur H, Iyer M, Vellingiri B (2024). Midbrain organoids for Parkinson’s disease (PD) - A powerful tool to understand the disease pathogenesis. Life Sci.

[R14] Bagley JA, Reumann D, Bian S, Lévi-Strauss J, Knoblich JA (2017). Fused cerebral organoids model interactions between brain regions. Nat Methods.

[R15] Bai Y, Wang Z, He X, Zhu Y, Xu X, Yang H, Mei G, Chen S, Ma B, Zhu R (2024). Application of bioactive materials for osteogenic function in bone tissue engineering. Small Methods.

[R16] Bak A, Koch H, van Loo KMJ, Schmied K, Gittel B, Weber Y, Ort J, Schwarz N, Tauber SC, Wuttke TV, Delev D (2024). Human organotypic brain slice cultures: a detailed and improved protocol for preparation and long-term maintenance. J Neurosci Methods.

[R17] Bao Z, Fang K, Miao Z, Li C, Yang C, Yu Q, Zhang C, Miao Z, Liu Y, Ji J (2021). Human cerebral organoid implantation alleviated the neurological deficits of traumatic brain injury in mice. Oxid Med Cell Longev.

[R18] Barker N, Huch M, Kujala P, van de Wetering M, Snippert HJ, van Es JH, Sato T, Stange DE, Begthel H, van den Born M, Danenberg E, van den Brink S, Korving J, Abo A, Peters PJ, Wright N, Poulsom R, Clevers H (2010). Lgr5(+ve) stem cells drive self-renewal in the stomach and build long-lived gastric units in vitro. Cell Stem Cell.

[R19] Barnhart AJ, Dierickx K (2022). The many moral matters of organoid models: a systematic review of reasons. Med Health Care Philos.

[R20] Barnhart AJ, Dierickx K (2023). Moving beyond the moral status of organoid-entities. Bioethics.

[R21] Berger E, Magliaro C, Paczia N, Monzel AS, Antony P, Linster CL, Bolognin S, Ahluwalia A, Schwamborn JC (2018). Millifluidic culture improves human midbrain organoid vitality and differentiation. Lab Chip.

[R22] Bian S, Repic M, Guo Z, Kavirayani A, Burkard T, Bagley JA, Krauditsch C, Knoblich JA (2018). Genetically engineered cerebral organoids model brain tumor formation. Nat Methods.

[R23] Birey F, Andersen J, Makinson CD, Islam S, Wei W, Huber N, Fan HC, Metzler KRC, Panagiotakos G, Thom N, O’Rourke NA, Steinmetz LM, Bernstein JA, Hallmayer J, Huguenard JR, Paşca SP (2017). Assembly of functionally integrated human forebrain spheroids. Nature.

[R24] Birey F, Li MY, Gordon A, Thete MV, Valencia AM, Revah O, Paşca AM, Geschwind DH, Paşca SP (2022). Dissecting the molecular basis of human interneuron migration in forebrain assembloids from Timothy syndrome. Cell Stem Cell.

[R25] Birtele M, Del Dosso A, Xu T, Nguyen T, Wilkinson B, Hosseini N, Nguyen S, Urenda JP, Knight G, Rojas C, Flores I, Atamian A, Moore R, Sharma R, Pirrotte P, Ashton RS, Huang EJ, Rumbaugh G, Coba MP, Quadrato G (2023). Non-synaptic function of the autism spectrum disorder-associated gene SYNGAP1 in cortical neurogenesis. Nat Neurosci.

[R26] Blair JD, Hockemeyer D, Bateup HS (2018). Genetically engineered human cortical spheroid models of tuberous sclerosis. Nat Med.

[R27] Boers SN, de Winter-de Groot KM, Noordhoek J, Gulmans V, van der Ent CK, van Delden JJM, Bredenoord AL (2018). Mini-guts in a dish: Perspectives of adult Cystic Fibrosis (CF) patients and parents of young CF patients on organoid technology. J Cyst Fibros.

[R28] Boussaad I (2020). A patient-based model of RNA mis-splicing uncovers treatment targets in Parkinson’s disease. Sci Transl Med.

[R29] Boyd JL (2024). Moral considerability of brain organoids from the perspective of computational architecture. Oxford Open Neurosci.

[R30] Bredenoord AL, Clevers H, Knoblich JA (2017). Human tissues in a dish: The research and ethical implications of organoid technology. Science.

[R31] Brown R, Lau H, LeDoux JE (2019). Understanding the higher-order approach to consciousness. Trends Cogn Sci.

[R32] Buonfiglioli A, Kübler R, Missall R, De Jong R, Chan S, Haage V, Wendt S, Lin AJ, Mattei D, Graziani M, Latour B, Gigase F, Chiu R, Zhang Y, Nygaard HB, De Jager PL, De Witte LD (2025). A microglia-containing cerebral organoid model to study early life immune challenges. Brain Behav Immun.

[R33] Busek M, Aizenshtadt A, Koch T, Frank A, Delon L, Martinez MA, Golovin A, Dumas C, Stokowiec J, Gruenzner S, Melum E, Krauss S (2023). Pump-less, recirculating organ-on-a-chip (rOoC) platform. Lab Chip.

[R34] Cakir B (2019). Engineering of human brain organoids with a functional vascular-like system. Nat Methods.

[R35] Cao SY, Yang D, Huang ZQ, Lin YH, Wu HY, Chang L, Luo CX, Xu Y, Liu Y, Zhu DY (2023). Cerebral organoids transplantation repairs infarcted cortex and restores impaired function after stroke. NPJ Regen Med.

[R36] Cao SY, Tao MD, Lou SN, Yang D, Lin YH, Wu HY, Chang L, Luo CX, Xu Y, Liu Y, Zhu DY (2023). Functional reconstruction of the impaired cortex and motor function by hMGEOs transplantation in stroke. Biochem Biophys Res Commun.

[R37] Carvalho MR, Yan LP, Li B, Zhang CH, He YL, Reis RL, Oliveira JM (2023). Gastrointestinal organs and organoids-on-a-chip: advances and translation into the clinics. Biofabrication.

[R38] Castiglione H, Vigneron PA, Baquerre C, Yates F, Rontard J, Honegger T (2022). Human brain organoids-on-chip: advances, challenges, and perspectives for preclinical applications. Pharmaceutics.

[R39] Cavalcante BRR, Aragão-França LS, Sampaio GLA, Nonaka CKV, Oliveira MS, Campos GS, Sardi SI, Dias BRS, Menezes JPB, Rocha VPC, Rossi EA, Paredes BD, Martins GLS, Allahdadi KJ, Peixoto LR, Barbosa-Filho JM, Souza BSF, Soares MBP (2020). Betulinic acid exerts cytoprotective activity on Zika virus-infected neural progenitor cells. Front Cell Infect Microbiol.

[R40] Chacko L, Chaudhary A, Singh B, Dewanjee S, Kandimalla R (2023). CRISPR-Cas9 in Alzheimer’s disease: Therapeutic trends, modalities, and challenges. Drug Discov Today.

[R41] Chen M, Tian H, Huang G, Fang T, Lin X, Shan J, Cai Z, Chen G, Chen S, Chen C, Ping J, Cheng L, Chen C, Zhu J, Zhao F, Jiang D, Liu C, Huang G, Lin C, Zhuo C (2021). Calcium imaging reveals depressive- and manic-phase-specific brain neural activity patterns in a murine model of bipolar disorder: a pilot study. Transl Psychiatry.

[R42] Chen X, Sun G, Tian E, Zhang M, Davtyan H, Beach TG, Reiman EM, Blurton-Jones M, Holtzman DM, Shi Y (2021). Modeling sporadic Alzheimer’s disease in human brain organoids under serum exposure. Adv Sci (Weinh).

[R43] Chen X, Birey F, Li MY, Revah O, Levy R, Thete MV, Reis N, Kaganovsky K, Onesto M, Sakai N, Hudacova Z, Hao J, Meng X, Nishino S, Huguenard J, Pașca SP (2024). Antisense oligonucleotide therapeutic approach for Timothy syndrome. Nature.

[R44] Chen YC, Farzadfard F, Gharaei N, Chen WCW, Cao J, Lu TK (2017). Randomized CRISPR-Cas transcriptional perturbation screening reveals protective genes against alpha-synuclein toxicity. Mol Cell.

[R45] Cheyne I, Gopinath VS, Muppa N, Armas AE, Gil Agurto MS, Akula SA, Nagpal S, Yousaf MS, Haider A (2024). The neurological implications of COVID-19: A comprehensive narrative review. Cureus.

[R46] Chiaradia I, Imaz-Rosshandler I, Nilges BS, Boulanger J, Pellegrini L, Das R, Kashikar ND, Lancaster MA (2023). Tissue morphology influences the temporal program of human brain organoid development. Cell Stem Cell.

[R47] Chiola S, Edgar NU, Shcheglovitov A (2022). iPSC toolbox for understanding and repairing disrupted brain circuits in autism. Mol Psychiatry.

[R48] Chliara MA, Elezoglou S, Zergioti I (2022). Bioprinting on organ-on-chip: Development and applications. Biosensors (Basel).

[R49] Cho AN (2021). Microfluidic device with brain extracellular matrix promotes structural and functional maturation of human brain organoids. Nat Commun.

[R50] Conforti P, Besusso D, Bocchi VD, Faedo A, Cesana E, Rossetti G, Ranzani V, Svendsen CN, Thompson LM, Toselli M, Biella G, Pagani M, Cattaneo E (2018). Faulty neuronal determination and cell polarization are reverted by modulating HD early phenotypes. Proc Natl Acad Sci U S A.

[R51] Dai R, Chen W, Chen Y, Jin J, Zhang S, Zhang C, Liu J (2024). 3D bioprinting platform development for high-throughput cancer organoid models construction and drug evaluation. Biofabrication.

[R52] Daviaud N, Friedel RH, Zou H (2018). Vascularization and engraftment of transplanted human cerebral organoids in mouse cortex. eNeuro.

[R53] de Majo M, Koontz M, Marsan E, Salinas N, Ramsey A, Kuo YM, Seo K, Li H, Dräger N, Leng K, Gonzales SL, Kurnellas M, Miyaoka Y, Klim JR, Kampmann M, Ward ME, Huang EJ, Ullian EM (2023). Granulin loss of function in human mature brain organoids implicates astrocytes in TDP-43 pathology. Stem Cell Reports.

[R54] Dell’Amico C, Angulo Salavarria MM, Takeo Y, Saotome I, Dell’Anno MT, Galimberti M, Pellegrino E, Cattaneo E, Louvi A, Onorati M (2023). Microcephaly-associated protein WDR62 shuttles from the Golgi apparatus to the spindle poles in human neural progenitors. Elife.

[R55] DeLong MR, Wichmann T (2007). Circuits and circuit disorders of the basal ganglia. Arch Neurol.

[R56] Ding Z, Tang N, Huang J, Cao X, Wu S (2023). Global hotspots and emerging trends in 3D bioprinting research. Front Bioeng Biotechnol.

[R57] Dixon TA, Muotri AR (2023). Advancing preclinical models of psychiatric disorders with human brain organoid cultures. Mol Psychiatry.

[R58] Dong X, Xu SB, Chen X, Tao M, Tang XY, Fang KH, Xu M, Pan Y, Chen Y, He S, Liu Y (2021). Human cerebral organoids establish subcortical projections in the mouse brain after transplantation. Mol Psychiatry.

[R59] Douceau S, Deutsch Guerrero T, Ferent J (2023). Establishing Hedgehog gradients during neural development. Cells.

[R60] Duzagac F, Saorin G, Memeo L, Canzonieri V, Rizzolio F (2021). Microfluidic organoids-on-a-chip: Quantum leap in cancer research. Cancers (Basel).

[R61] Eiraku M, Watanabe K, Matsuo-Takasaki M, Kawada M, Yonemura S, Matsumura M, Wataya T, Nishiyama A, Muguruma K, Sasai Y (2008). Self-organized formation of polarized cortical tissues from ESCs and its active manipulation by extrinsic signals. Cell Stem Cell.

[R62] Evans M (1981). Origin of mouse embryonal carcinoma cells and the possibility of their direct isolation into tissue culture. J Reprod Fertil.

[R63] Fair SR (2023). Cerebral organoids containing an AUTS2 missense variant model microcephaly. Brain.

[R64] Fan C, Cai H, Zhang L, Wu X, Yan J, Jin L, Hu B, He J, Chen Y, Zhao Y, Dai J (2024). Constructing linear-oriented pre-vascularized human spinal cord tissues for spinal cord injury repair. Adv Healthc Mater.

[R65] Farisco M, Changeux JP (2023). About the compatibility between the perturbational complexity index and the global neuronal workspace theory of consciousness. Neurosci Conscious.

[R66] Fattorelli N, Martinez-Muriana A, Wolfs L, Geric I, De Strooper B, Mancuso R (2021). Stem-cell-derived human microglia transplanted into mouse brain to study human disease. Nat Protoc.

[R67] Feng L, Liu Y, Li P, Wan H, Deng X, Wang T, Fu H, Duan X (2024). Association between cerebrovascular disease and perioperative neurocognitive disorders: a retrospective cohort study. Int J Surg.

[R68] Fitzgerald MQ, Chu T, Puppo F, Blanch R, Chillón M, Subramaniam S, Muotri AR (2024). Generation of ‘semi-guided’ cortical organoids with complex neural oscillations. Nat Protoc.

[R69] Frisvold S, Coppola S, Ehrmann S, Chiumello D, Guérin C (2023). Respiratory challenges and ventilatory management in different types of acute brain-injured patients. Crit Care.

[R70] Goel K, Phillips HW, Chen JS, Ngo J, Edmonds B, Ha PX, Wang A, Weil A, Russell BE, Salamon N, Nariai H, Fallah A (2024). Hemispheric epilepsy surgery for hemimegalencephaly: The UCLA experience. Epilepsia.

[R71] Góralczyk-Bińkowska A, Szmajda-Krygier D, Kozłowska E (2022). The microbiota-gut-brain axis in psychiatric disorders. Int J Mol Sci.

[R72] Granton E (2024). Biofilm exopolysaccharides alter sensory-neuron-mediated sickness during lung infection. Cell.

[R73] Groveman BR, Ferreira NC, Foliaki ST, Walters RO, Winkler CW, Race B, Hughson AG, Zanusso G, Haigh CL (2021). Human cerebral organoids as a therapeutic drug screening model for Creutzfeldt-Jakob disease. Sci Reports.

[R74] Ham O, Jin YB, Kim J, Lee MO (2020). Blood vessel formation in cerebral organoids formed from human embryonic stem cells. Biochem Biophys Res Commun.

[R75] Han CZ (2023). Human microglia maturation is underpinned by specific gene regulatory networks. Immunity.

[R76] Hanseeuw BJ (2019). Association of amyloid and tau with cognition in preclinical Alzheimer disease: A longitudinal study. JAMA Neurol.

[R77] Harbuzariu A, Nti A, Harp KO, Cespedes JC, Driss A, Stiles JK (2022). Neuregulin-1/ErbB4 signaling modulates Plasmodium falciparum HRP2-induced damage to brain cortical organoids. iScience.

[R78] Haselager DR, Boers SN, Jongsma KR, Vinkers CH, Broekman ML, Bredenoord AL (2020). Breeding brains? Patients’ and laymen’s perspectives on cerebral organoids. Regen Med.

[R79] He X, Li X, Zhao J, Mu C, Mi H, Hu J, Shi F (2022). Improving the catalytic and mechanical performance of alginate catalyst through functionalization by aminopolycarboxylic acids. J Colloid Interface Sci.

[R80] Hilkens NA, Casolla B, Leung TW, de Leeuw FE (2024). Stroke. Lancet.

[R81] Hirota T, King BH (2023). Autism spectrum disorder: A review. JAMA.

[R82] Hong H, Yoon SB, Park JE, Lee JI, Kim HY, Nam HJ, Cho H (2023). MeCP2 dysfunction prevents proper BMP signaling and neural progenitor expansion in brain organoid. Ann Clin Transl Neurol.

[R83] Hosseini SM, Alizadeh A, Shahsavani N, Chopek J, Ahlfors JE, Karimi-Abdolrezaee S (2022). Suppressing CSPG/LAR/PTPσ axis facilitates neuronal replacement and synaptogenesis by human neural precursor grafts and improves recovery after spinal cord injury. J Neurosci.

[R84] Hu D, Cao Y, Cai C, Wang G, Zhou M, Peng L, Fan Y, Lai Q, Gao Z (2025). Establishment of human cerebral organoid systems to model early neural development and assess the central neurotoxicity of environmental toxins. Neural Regen Res.

[R85] Hu H, Wang C, Tao R, Liu B, Peng D, Chen Y, Zhang W (2024). Evidences of neurological injury caused by COVID-19 from glioma tissues and glioma organoids. CNS Neurosci Ther.

[R86] Huang G, Li Z, Liu X, Guan M, Zhou S, Zhong X, Zheng T, Xin D, Gu X, Mu D, Guo Y, Zhang L, Zhang L, Lu QR, He X (2024). DOR activation in mature oligodendrocytes regulates α-ketoglutarate metabolism leading to enhanced remyelination in aged mice. Nat Neurosci.

[R87] Huang S, Zhang Z, Cao J, Yu Y, Pei G (2022). Chimeric cerebral organoids reveal the essentials of neuronal and astrocytic APOE4 for Alzheimer’s tau pathology. Signal Transduct Target Ther.

[R88] Huang Y, Lee S, Liu W, Takayama S, Jia S (2023). OctoShaker: A versatile robotic biomechanical agitator for cellular and organoid research. Rev Sci Instrum.

[R89] Huang Y (2024). Loss-of-function in RBBP5 results in a syndromic neurodevelopmental disorder associated with microcephaly. Genet Med.

[R90] Hwang I, Kim BS, Lee HY, Cho SW, Lee SE, Ahn JY (2024). PA2G4/EBP1 ubiquitination by PRKN/PARKIN promotes mitophagy protecting neuron death in cerebral ischemia. Autophagy.

[R91] Hyun I (2022). How collaboration between bioethicists and neuroscientists can advance research. Nat Neurosci.

[R92] Klincewicz M, Cheng T, Schmitz M, Sebastián MÁ, Snyder JS, IIT-Concerned (2025). What makes a theory of consciousness unscientific?. Nat Neurosci.

[R93] Ijezie EC, O’Dowd JM, Kuan MI, Faeth AR, Fortunato EA (2023). HCMV infection reduces Nidogen-1 expression, contributing to impaired neural rosette development in brain organoids. J Virol.

[R94] Jackson CB, Farzan M, Chen B, Choe H (2022). Mechanisms of SARS-CoV-2 entry into cells. Nat Rev Mol Cell Biol.

[R95] Jacques TS, Swales A, Brzozowski MJ, Henriquez NV, Linehan JM, Mirzadeh Z, C OM, Naumann H, Alvarez-Buylla A, Brandner S (2010). Combinations of genetic mutations in the adult neural stem cell compartment determine brain tumour phenotypes. EMBO J.

[R96] Janssens S, Schotsaert M, Manganaro L, Dejosez M, Simon V, García-Sastre A, Zwaka TP (2019). FACS-mediated isolation of neuronal cell populations from virus-infected human embryonic stem cell-derived cerebral organoid cultures. Curr Protoc Stem Cell Biol.

[R97] Jarazo J (2022). Parkinson’s disease phenotypes in patient neuronal cultures and brain organoids improved by 2-hydroxypropyl-β-cyclodextrin treatment. Mov Disord.

[R98] Jgamadze D (2023). Structural and functional integration of human forebrain organoids with the injured adult rat visual system. Cell Stem Cell.

[R99] Ji Q, Lv Y, Hu B, Su Y, Shaikh II, Zhu X (2024). Study on the therapeutic potential of induced neural stem cells for Alzheimer’s disease in mice. Biol Res.

[R100] Ji S (2023). Pharmaco-proteogenomic characterization of liver cancer organoids for precision oncology. Sci Transl Med.

[R101] Jiang S, Zhao H, Zhang W, Wang J, Liu Y, Cao Y, Zheng H, Hu Z, Wang S, Zhu Y, Wang W, Cui S, Lobie PE, Huang L, Ma S (2020). An automated organoid platform with inter-organoid homogeneity and inter-patient heterogeneity. Cell Rep Med.

[R102] Jin M, Xu R, Wang L, Alam MM, Ma Z, Zhu S, Martini AC, Jadali A, Bernabucci M, Xie P, Kwan KY, Pang ZP, Head E, Liu Y, Hart RP, Jiang P (2022). Type-I-interferon signaling drives microglial dysfunction and senescence in human iPSC models of Down syndrome and Alzheimer’s disease. Cell Stem Cell.

[R103] Jiu J, Liu H, Li D, Li J, Liu L, Yang W, Yan L, Li S, Zhang J, Li X, Li JJ, Wang B (2024). 3D bioprinting approaches for spinal cord injury repair. Biofabrication.

[R104] Jo J (2016). Midbrain-like organoids from human pluripotent stem cells contain functional dopaminergic and neuromelanin-producing neurons. Cell Stem Cell.

[R105] Jo J, Yang L, Tran HD, Yu W, Sun AX, Chang YY, Jung BC, Lee SJ, Saw TY, Xiao B, Khoo ATT, Yaw LP, Xie JJ, Lokman H, Ong WY, Lim GGY, Lim KL, Tan EK, Ng HH, Je HS (2021). Lewy Body-like Inclusions in Human Midbrain Organoids Carrying Glucocerebrosidase and α-Synuclein Mutations. Ann Neurol.

[R106] Ju D, Dong C (2024). The combined application of stem cells and three-dimensional bioprinting scaffolds for the repair of spinal cord injury. Neural Regen Res.

[R107] Karvas RM, Khan SA, Verma S, Yin Y, Kulkarni D, Dong C, Park KM, Chew B, Sane E, Fischer LA, Kumar D, Ma L, Boon ACM, Dietmann S, Mysorekar IU, Theunissen TW (2022). Stem-cell-derived trophoblast organoids model human placental development and susceptibility to emerging pathogens. Cell Stem Cell.

[R108] Kataoka M, Ishida S, Kobayashi C, Lee TL, Sawai T (2025). Evaluating neuroprivacy concerns in human brain organoid research. Trends Biotechnol.

[R109] Kathuria A, Lopez-Lengowski K, Vater M, McPhie D, Cohen BM, Karmacharya R (2020). Transcriptome analysis and functional characterization of cerebral organoids in bipolar disorder. Genome Med.

[R110] Kathuria A, Lopez-Lengowski K, Jagtap SS, McPhie D, Perlis RH, Cohen BM, Karmacharya R (2020). Transcriptomic landscape and functional characterization of induced pluripotent stem cell-derived cerebral organoids in schizophrenia. JAMA Psychiatry.

[R111] Kathuria A, Lopez-Lengowski K, McPhie D, Cohen BM, Karmacharya R (2023). Disease-specific differences in gene expression, mitochondrial function and mitochondria-endoplasmic reticulum interactions in iPSC-derived cerebral organoids and cortical neurons in schizophrenia and bipolar disorder. Discov Ment Health.

[R112] Kawakita S, Mandal K, Mou L, Mecwan MM, Zhu Y, Li S, Sharma S, Hernandez AL, Nguyen HT, Maity S, de Barros NR, Nakayama A, Bandaru P, Ahadian S, Kim HJ, Herculano RD, Holler E, Jucaud V, Dokmeci MR, Khademhosseini A (2022). Organ-on-a-chip models of the blood-brain barrier: Recent advances and future prospects. Small.

[R113] Kelava I, Chiaradia I, Pellegrini L, Kalinka AT, Lancaster MA (2022). Androgens increase excitatory neurogenic potential in human brain organoids. Nature.

[R114] Kelley KW, Revah O, Gore F, Kaganovsky K, Chen X, Deisseroth K, Pașca SP (2024). Host circuit engagement of human cortical organoids transplanted in rodents. Nat Protoc.

[R115] Kennedy H, Wianny F, Dehay C (2021). Determinants of primate neurogenesis and the deployment of top-down generative networks in the cortical hierarchy. Curr Opin Neurobiol.

[R116] Kim H, Park HJ, Choi H, Chang Y, Park H, Shin J, Kim J, Lengner CJ, Lee YK, Kim J (2019). Modeling G2019S-LRRK2 sporadic Parkinson’s disease in 3D midbrain organoids. Stem Cell Reports.

[R117] Kim JT, Kim TY, Youn DH, Han SW, Park CH, Lee Y, Jung H, Rhim JK, Park JJ, Ahn JH, Kim HC, Cho SM, Jeon JP (2022). Human embryonic stem cell-derived cerebral organoids for treatment of mild traumatic brain injury in a mouse model. Biochem Biophys Res Commun.

[R118] Kitahara T, Sakaguchi H, Morizane A, Kikuchi T, Miyamoto S, Takahashi J (2020). Axonal extensions along corticospinal tracts from transplanted human cerebral organoids. Stem Cell Reports.

[R119] Koh I, Hagiwara M (2024). Modular tissue-in-a-CUBE platform to model blood-brain barrier (BBB) and brain interaction. Commun Biol.

[R120] Kong W, Frouard J, Xie G, Corley MJ, Helmy E, Zhang G, Schwarzer R, Montano M, Sohn P, Roan NR, Ndhlovu LC, Gan L, Greene WC (2024). Neuroinflammation generated by HIV-infected microglia promotes dysfunction and death of neurons in human brain organoids. PNAS Nexus.

[R121] Koplin JJ, Savulescu J (2019). Moral limits of brain organoid research. J Law Med Ethics.

[R122] Koplin JJ (2024). Weighing the moral status of brain organoids and research animals. Bioethics.

[R123] Kowalski RG, Hammond FM, Weintraub AH, Nakase-Richardson R, Zafonte RD, Whyte J, Giacino JT (2021). Recovery of consciousness and functional outcome in moderate and severe traumatic brain injury. JAMA Neurol.

[R124] Kratochvil MJ, Seymour AJ, Li TL, Paşca SP, Kuo CJ, Heilshorn SC (2019). Engineered materials for organoid systems. Nat Rev Mater.

[R125] Kuehner JN, Chen J, Bruggeman EC, Wang F, Li Y, Xu C, McEachin ZT, Li Z, Chen L, Hales CM, Wen Z, Yang J, Yao B (2021). 5-hydroxymethylcytosine is dynamically regulated during forebrain organoid development and aberrantly altered in Alzheimer’s disease. Cell Rep.

[R126] Lana-Elola E, Aoidi R, Llorian M, Gibbins D, Buechsenschuetz C, Bussi C, Flynn H, Gilmore T, Watson-Scales S, Haugsten Hansen M, Hayward D, Song OR, Brault V, Herault Y, Deau E, Meijer L, Snijders AP, Gutierrez MG, Fisher EMC, Tybulewicz VLJ (2024). Increased dosage of DYRK1A leads to congenital heart defects in a mouse model of Down syndrome. Sci Transl Med.

[R127] Lancaster MA, Renner M, Martin CA, Wenzel D, Bicknell LS, Hurles ME, Homfray T, Penninger JM, Jackson AP, Knoblich JA (2013). Cerebral organoids model human brain development and microcephaly. Nature.

[R128] Lancaster MA, Knoblich JA (2014). Generation of cerebral organoids from human pluripotent stem cells. Nat Protoc.

[R129] Lancaster MA, Corsini NS, Wolfinger S, Gustafson EH, Phillips AW, Burkard TR, Otani T, Livesey FJ, Knoblich JA (2017). Guided self-organization and cortical plate formation in human brain organoids. Nat Biotechnol.

[R130] Lavazza A (2020). Human cerebral organoids and consciousness: a double-edged sword. Monash Bioeth Rev.

[R131] Lee H, Thacker S, Sarn N, Dutta R, Eng C (2019). Constitutional mislocalization of Pten drives precocious maturation in oligodendrocytes and aberrant myelination in model of autism spectrum disorder. Transl Psychiatry.

[R132] Lénárt N, Cserép C, Császár E, Pósfai B, Dénes Á (2024). Microglia-neuron-vascular interactions in ischemia. Glia.

[R133] Lensink MA, Jongsma KR, Boers SN, Noordhoek JJ, Beekman JM, Bredenoord AL (2020). Responsible use of organoids in precision medicine: the need for active participant involvement. Development.

[R134] Lensink MA, Boers SN, VA MG, Jongsma KR, Bredenoord AL (2021). Mini-gut feelings: perspectives of people with cystic fibrosis on the ethics and governance of organoid biobanking. Personalized Med.

[R135] Lensink MA, Boers SN, Jongsma KR, Carter SE, van der Ent CK, Bredenoord AL (2021). Organoids for personalized treatment of cystic fibrosis: Professional perspectives on the ethics and governance of organoid biobanking. J Cyst Fibros.

[R136] Li C (2017). 25-Hydroxycholesterol protects host against Zika virus infection and its associated microcephaly in a mouse model. Immunity.

[R137] Li C, Fleck JS, Martins-Costa C, Burkard TR, Themann J, Stuempflen M, Peer AM, Vertesy Á, Littleboy JB, Esk C, Elling U, Kasprian G, Corsini NS, Treutlein B, Knoblich JA (2023). Single-cell brain organoid screening identifies developmental defects in autism. Nature.

[R138] Li J, Chu J, Lui VCH, Chen S, Chen Y, Tam PKH (2022). Bioengineering liver organoids for diseases modelling and transplantation. Bioengineering (Basel).

[R139] Li K, Wang Z (2023). lncRNA NEAT1: Key player in neurodegenerative diseases. Ageing Res Rev.

[R140] Li ML, Aggeler J, Farson DA, Hatier C, Hassell J, Bissell MJ (1987). Influence of a reconstituted basement membrane and its components on casein gene expression and secretion in mouse mammary epithelial cells. Proc Natl Acad Sci U S A.

[R141] Li R, Sun L, Fang A, Li P, Wu Q, Wang X (2017). Recapitulating cortical development with organoid culture in vitro and modeling abnormal spindle-like (ASPM related primary) microcephaly disease. Protein Cell.

[R142] Li XH, Guo D, Chen LQ, Chang ZH, Shi JX, Hu N, Chen C, Zhang XW, Bao SQ, Chen MM, Ming D (2024). Low-intensity ultrasound ameliorates brain organoid integration and rescues microcephaly deficits. Brain.

[R143] Li Z, Xu J, Lang Y, Wu X, Hu S, Samrat SK, Tharappel AM, Kuo L, Butler D, Song Y, Zhang QY, Zhou J, Li H (2022). In vitro and in vivo characterization of erythrosin B and derivatives against Zika virus. Acta Pharm Sin B.

[R144] Liang KX (2024). The application of brain organoid for drug discovery in mitochondrial diseases. Int J Biochem Cell Biol.

[R145] Lieberthal B, Allan B, De Urioste-Stone S, Mackay A, Soliman A, Wang S, Gardner AM (2024). The effects of seasonal human mobility and Aedes aegypti habitat suitability on Zika virus epidemic severity in Colombia. PLoS Negl Trop Dis.

[R146] Lin YT (2018). APOE4 causes widespread molecular and cellular alterations associated with Alzheimer’s disease phenotypes in human iPSC-derived brain cell types. Neuron.

[R147] Linkous A, Balamatsias D, Snuderl M, Edwards L, Miyaguchi K, Milner T, Reich B, Cohen-Gould L, Storaska A, Nakayama Y, Schenkein E, Singhania R, Cirigliano S, Magdeldin T, Lin Y, Nanjangud G, Chadalavada K, Pisapia D, Liston C, Fine HA (2019). Modeling patient-derived glioblastoma with cerebral organoids. Cell Rep.

[R148] Lisowski P (2024). Mutant huntingtin impairs neurodevelopment in human brain organoids through CHCHD2-mediated neurometabolic failure. Nat Commun.

[R149] Liu C, Fu Z, Wu S, Wang X, Zhang S, Chu C, Hong Y, Wu W, Chen S, Jiang Y, Wu Y, Song Y, Liu Y, Guo X (2022). Mitochondrial HSF1 triggers mitochondrial dysfunction and neurodegeneration in Huntington’s disease. EMBO Mol Med.

[R150] Liu M, Huang J, Liu T, Yuan J, Lv C, Sha Z, Wu C, Jiang W, Liu X, Nie M, Chen Y, Dong S, Qian Y, Gao C, Fan Y, Wu D, Jiang R (2023). Exogenous interleukin 33 enhances the brain’s lymphatic drainage and toxic protein clearance in acute traumatic brain injury mice. Acta Neuropathol Commun.

[R151] Liu M, Zhang W, Han S, Zhang D, Zhou X, Guo X, Chen H, Wang H, Jin L, Feng S, Wei Z (2024). Multifunctional conductive and electrogenic hydrogel repaired spinal cord injury via immunoregulation and enhancement of neuronal differentiation. Adv Mater.

[R152] Liu X, Wu C, Zhang Y, Chen S, Ding J, Chen Z, Wu K, Wu X, Zhou T, Zeng M, Wei D, Sun J, Fan H, Zhou L (2023). Hyaluronan-based hydrogel integrating exosomes for traumatic brain injury repair by promoting angiogenesis and neurogenesis. Carbohydr Polym.

[R153] Lorenzetti S, Mikhail M, Di Benedetto L, Dellambra E, Failla CM, Giannitelli SM, Levato R, Longoni A, Bartolocci V, Lulli D, Raniolo S, De Angelis I (2024). 3D bioprinting of human skin and squamous cell tumors (SCCs) as advanced models for precision medicine (BIOSQIN). Altex.

[R154] Lovell-Badge R (2021). ISSCR Guidelines for Stem Cell Research and Clinical Translation: The 2021 update. Stem Cell Reports.

[R155] Lu K, Hong Y, Tao M, Shen L, Zheng Z, Fang K, Yuan F, Xu M, Wang C, Zhu D, Guo X, Liu Y (2023). Depressive patient-derived GABA interneurons reveal abnormal neural activity associated with HTR2C. EMBO Mol Med.

[R156] Lu S, Zhu X, Zeng P, Hu L, Huang Y, Guo X, Chen Q, Wang Y, Lai L, Xue A, Wang Y, Wang Z, Song W, Liu Q, Bian G, Li J, Bu Q, Cen X (2024). Exposure to PFOA, PFOS, and PFHxS induces Alzheimer’s disease-like neuropathology in cerebral organoids. Environ Pollut.

[R157] Lv S, Luo C (2025). Ferroptosis in schizophrenia: Mechanisms and therapeutic potentials (Review). Mol Med Rep.

[R158] Madhavan M, Nevin ZS, Shick HE, Garrison E, Clarkson-Paredes C, Karl M, Clayton BLL, Factor DC, Allan KC, Barbar L, Jain T, Douvaras P, Fossati V, Miller RH, Tesar PJ (2018). Induction of myelinating oligodendrocytes in human cortical spheroids. Nat Methods.

[R159] Maharjan S, Ma C, Singh B, Kang H, Orive G, Yao J, Shrike Zhang Y (2024). Advanced 3D imaging and organoid bioprinting for biomedical research and therapeutic applications. Adv Drug Deliv Rev.

[R160] Majc B, Habič A, Malavolta M, Vittori M, Porčnik A, Bošnjak R, Mlakar J, Matjašič A, Zupan A, Vidmar MS, Turnšek TL, Sadikov A, Breznik B, Novak M (2024). Patient-derived tumor organoids mimic treatment-induced DNA damage response in glioblastoma. iScience.

[R161] Mangena V, Chanoch-Myers R, Sartore R, Paulsen B, Gritsch S, Weisman H, Hara T, Breakefield XO, Breyne K, Regev A, Chung K, Arlotta P, Tirosh I, Suva ML (2025). Glioblastoma-cortical organoids recapitulate cell state heterogeneity and intercellular transfer. Cancer Discov.

[R162] Mansour AA, Gonçalves JT, Bloyd CW, Li H, Fernandes S, Quang D, Johnston S, Parylak SL, Jin X, Gage FH (2018). An in vivo model of functional and vascularized human brain organoids. Nat Biotechnol.

[R163] Manz KM, Siemann JK, McMahon DG, Grueter BA (2021). Patch-clamp and multi-electrode array electrophysiological analysis in acute mouse brain slices. STAR Protoc.

[R164] Marchetto MC (2017). Altered proliferation and networks in neural cells derived from idiopathic autistic individuals. Mol Psychiatry.

[R165] Mariani J, Simonini MV, Palejev D, Tomasini L, Coppola G, Szekely AM, Horvath TL, Vaccarino FM (2012). Modeling human cortical development in vitro using induced pluripotent stem cells. Proc Natl Acad Sci U S A.

[R166] Mariani J, Coppola G, Zhang P, Abyzov A, Provini L, Tomasini L, Amenduni M, Szekely A, Palejev D, Wilson M, Gerstein M, Grigorenko EL, Chawarska K, Pelphrey KA, Howe JR, Vaccarino FM (2015). FOXG1-dependent dysregulation of GABA/glutamate neuron differentiation in autism spectrum disorders. Cell.

[R167] Martin GR (1981). Isolation of a pluripotent cell line from early mouse embryos cultured in medium conditioned by teratocarcinoma stem cells. Proc Natl Acad Sci U S A.

[R168] Marton RM, Miura Y, Sloan SA, Li Q, Revah O, Levy RJ, Huguenard JR, Pașca SP (2019). Differentiation and maturation of oligodendrocytes in human three-dimensional neural cultures. Nat Neurosci.

[R169] Marton RM, Pașca SP (2020). Organoid and assembloid technologies for investigating cellular crosstalk in human brain development and disease. Trends Cell Biol.

[R170] Mätlik K, Baffuto M, Kus L, Deshmukh AL, Davis DA, Paul MR, Carroll TS, Caron MC, Masson JY, Pearson CE, Heintz N (2024). Cell-type-specific CAG repeat expansions and toxicity of mutant Huntingtin in human striatum and cerebellum. Nat Genet.

[R171] Mesci P, de Souza JS, Martin-Sancho L, Macia A, Saleh A, Yin X, Snethlage C, Adams JW, Avansini SH, Herai RH, Almenar-Queralt A, Pu Y, Szeto RA, Goldberg G, Bruck PT, Papes F, Chanda SK, Muotri AR (2022). SARS-CoV-2 infects human brain organoids causing cell death and loss of synapses that can be rescued by treatment with Sofosbuvir. PLoS Biol.

[R172] Mestas J, Hughes CC (2004). Of mice and not men: differences between mouse and human immunology. J Immunol.

[R173] Meyer K, Ling KH, Yeo PL, Spathopoulou A, Drake D, Choi J, Aron L, Garcia-Corral M, Ko T, Lee EA, Tam JM, Perlis RH, Church GM, Tsai LH, Yankner BA (2024). Impaired neural stress resistance and loss of REST in bipolar disorder. Mol Psychiatry.

[R174] Miller P, Patel SR, Skinner R, Dignan F, Richter A, Jeffery K, Khan A, Heath PT, Clark A, Orchard K, Snowden JA, de Silva TI (2023). Joint consensus statement on the vaccination of adult and paediatric haematopoietic stem cell transplant recipients: Prepared on behalf of the British society of blood and marrow transplantation and cellular therapy (BSBMTCT), the Children’s cancer and Leukaemia Group (CCLG), and British Infection Association (BIA). J Infect.

[R175] Miura Y, Li MY, Birey F, Ikeda K, Revah O, Thete MV, Park JY, Puno A, Lee SH, Porteus MH, Pașca SP (2020). Generation of human striatal organoids and cortico-striatal assembloids from human pluripotent stem cells. Nat Biotechnol.

[R176] Miura Y, Li MY, Revah O, Yoon SJ, Narazaki G, Pașca SP (2022). Engineering brain assembloids to interrogate human neural circuits. Nat Protoc.

[R177] Montagne A, Barnes SR, Sweeney MD, Halliday MR, Sagare AP, Zhao Z, Toga AW, Jacobs RE, Liu CY, Amezcua L, Harrington MG, Chui HC, Law M, Zlokovic BV (2015). Blood-brain barrier breakdown in the aging human hippocampus. Neuron.

[R178] Monzel AS, Smits LM, Hemmer K, Hachi S, Moreno EL, van Wuellen T, Jarazo J, Walter J, Brüggemann I, Boussaad I, Berger E, Fleming RMT, Bolognin S, Schwamborn JC (2017). Derivation of human midbrain-specific organoids from neuroepithelial stem cells. Stem Cell Reports.

[R179] Morris HR, Spillantini MG, Sue CM, Williams-Gray CH (2024). The pathogenesis of Parkinson’s disease. Lancet.

[R180] Mrza MA, He J, Wang Y (2024). Integration of iPSC-derived microglia into brain organoids for neurological research. Int J Mol Sci.

[R181] Mühlebner A (2016). Novel histopathological patterns in cortical tubers of epilepsy surgery patients with tuberous sclerosis complex. PLoS One.

[R182] Mulder LA, Depla JA, Sridhar A, Wolthers K, Pajkrt D, Vieira de Sá R (2023). A beginner’s guide on the use of brain organoids for neuroscientists: a systematic review. Stem Cell Res Ther.

[R183] Mumby P, Adams W, Smith S, Rao M, Stiff P (2024). Pharmacotherapy for the prevention of depression and behavioral side effects in hematopoietic stem cell transplantation patients. Transpl Cell Ther.

[R184] Nge PN, Rogers CI, Woolley AT (2013). Advances in microfluidic materials, functions, integration, and applications. Chem Rev.

[R185] Nickl V, Eck J, Goedert N, Hübner J, Nerreter T, Hagemann C, Ernestus RI, Schulz T, Nickl RC, Keßler AF, Löhr M, Rosenwald A, Breun M, Monoranu CM (2023). Characterization and optimization of the tumor microenvironment in patient-derived organotypic slices and organoid models of glioblastoma. Cancers (Basel).

[R186] Nie YZ, Zheng YW, Ogawa M, Miyagi E, Taniguchi H (2018). Human liver organoids generated with single donor-derived multiple cells rescue mice from acute liver failure. Stem Cell Res Ther.

[R187] Ning W, Lv S, Wang Q, Xu Y (2025). The pivotal role of microglia in injury and the prognosis of subarachnoid hemorrhage. Neural Regen Res.

[R188] Niu W, Yu S, Li X, Wang Z, Chen R, Michalski C, Jahangiri A, Zohdy Y, Chern JJ, Whitworth TJ, Wang J, Xu J, Zhou Y, Qin Z, Li B, Gambello MJ, Peng J, Wen Z (2024). Longitudinal multi-omics reveals pathogenic TSC2 variants disrupt developmental trajectories of human cortical organoids derived from Tuberous Sclerosis Complex. bioRxiv [Preprint].

[R189] Nolan J, Pearce OMT, Screen HRC, Knight MM, Verbruggen SW (2023). Organ-on-a-chip and microfluidic platforms for oncology in the UK. Cancers (Basel).

[R190] Notaras M, Lodhi A, Dündar F, Collier P, Sayles NM, Tilgner H, Greening D, Colak D (2022). Schizophrenia is defined by cell-specific neuropathology and multiple neurodevelopmental mechanisms in patient-derived cerebral organoids. Mol Psychiatry.

[R191] Numakawa T, Kajihara R (2023). Neurotrophins and other growth factors in the pathogenesis of Alzheimer’s disease. Life (Basel).

[R192] Ogawa J, Pao GM, Shokhirev MN, Verma IM (2018). Glioblastoma model using human cerebral organoids. Cell Rep.

[R193] Olatunji IE, Rauch J, Katzensteiner M, Khosla M (2024). A review of anonymization for healthcare data. Big Data.

[R194] Osete JR, Akkouh IA, Ievglevskyi O, Vandenberghe M, de Assis DR, Ueland T, Kondratskaya E, Holen B, Szabo A, Hughes T, Smeland OB, Steen VM, Andreassen OA, Djurovic S (2023). Transcriptional and functional effects of lithium in bipolar disorder iPSC-derived cortical spheroids. Mol Psychiatry.

[R195] Ozgun A, Lomboni DJ, Aylsworth A, Macdonald A, Staines WA, Martina M, Schlossmacher MG, Tauskela JS, Woulfe J, Variola F (2024). Unraveling the assembloid: Real-time monitoring of dopaminergic neurites in an inter-organoid pathway connecting midbrain and striatal regions. Materials Today Bio.

[R196] Page CE, Epperson CN, Novick AM, Duffy KA, Thompson SM (2024). Beyond the serotonin deficit hypothesis: communicating a neuroplasticity framework of major depressive disorder. Mol Psychiatry.

[R197] Palasantzas V, Tamargo-Rubio I, Le K, Slager J, Wijmenga C, Jonkers IH, Kumar V, Fu J, Withoff S (2023). iPSC-derived organ-on-a-chip models for personalized human genetics and pharmacogenomics studies. Trends Genet.

[R198] Pallavicini G (2024). Modeling primary microcephaly with human brain organoids reveals fundamental roles of CIT kinase activity. J Clin Invest.

[R199] Pamies D, Block K, Lau P, Gribaldo L, Pardo CA, Barreras P, Smirnova L, Wiersma D, Zhao L, Harris G, Hartung T, Hogberg HT (2018). Rotenone exerts developmental neurotoxicity in a human brain spheroid model. Toxicol Appl Pharmacol.

[R200] Park DS (2023). iPS-cell-derived microglia promote brain organoid maturation via cholesterol transfer. Nature.

[R201] Park JC, Jang SY, Lee D, Lee J, Kang U, Chang H, Kim HJ, Han SH, Seo J, Choi M, Lee DY, Byun MS, Yi D, Cho KH, Mook-Jung I (2021). A logical network-based drug-screening platform for Alzheimer’s disease representing pathological features of human brain organoids. Nat Commun.

[R202] Pașca SP, Arlotta P, Bateup HS, Camp JG, Cappello S, Gage FH, Knoblich JA, Kriegstein AR, Lancaster MA, Ming GL, Muotri AR, Park IH, Reiner O, Song H, Studer L, Temple S, Testa G, Treutlein B, Vaccarino FM (2022). A nomenclature consensus for nervous system organoids and assembloids. Nature.

[R203] Patton MH, Thomas KT, Bayazitov IT, Newman KD, Kurtz NB, Robinson CG, Ramirez CA, Trevisan AJ, Bikoff JB, Peters ST, Pruett-Miller SM, Jiang Y, Schild AB, Nityanandam A, Zakharenko SS (2024). Synaptic plasticity in human thalamocortical assembloids. Cell Rep.

[R204] Paulsen B (2022). Autism genes converge on asynchronous development of shared neuron classes. Nature.

[R205] Pavon N, Diep K, Yang F, Sebastian R, Martinez-Martin B, Ranjan R, Sun Y, Pak C (2024). Patterning ganglionic eminences in developing human brain organoids using a morphogen-gradient-inducing device. Cell Rep Methods.

[R206] Pellegrini L, Albecka A, Mallery DL, Kellner MJ, Paul D, Carter AP, James LC, Lancaster MA (2020). SARS-CoV-2 infects the brain choroid plexus and disrupts the blood-CSF barrier in human brain organoids. Cell Stem Cell.

[R207] Peng K, Xie W, Wang T, Li Y, de Dieu Habimana J, Amissah OB, Huang J, Chen Y, Ni B, Li Z (2023). HIF-1α promotes kidney organoid vascularization and applications in disease modeling. Stem Cell Res Ther.

[R208] Phalnikar K, Srividya M, Mythri SV, Vasavi NS, Ganguly A, Kumar A, S P, Kalia K, Mishra SS, Dhanya SK, Paul P, Holla B, Ganesh S, Reddy PC, Sud R, Viswanath B, Muralidharan B (2024). Altered neuroepithelial morphogenesis and migration defects in iPSC-derived cerebral organoids and 2D neural stem cells in familial bipolar disorder. Oxford Open Neurosci.

[R209] Pigoni M, Uzquiano A, Paulsen B, Kedaigle AJ, Yang SM, Symvoulidis P, Adiconis X, Velasco S, Sartore R, Kim K, Tucewicz A, Tropp SY, Tsafou K, Jin X, Barrett L, Chen F, Boyden ES, Regev A, Levin JZ, Arlotta P (2023). Cell-type specific defects in PTEN-mutant cortical organoids converge on abnormal circuit activity. Hum Mol Genet.

[R210] Pode-Shakked N, Devarajan P (2022). Human stem cell and organoid models to advance acute kidney injury diagnostics and therapeutics. Int J Mol Sci.

[R211] Popova G, Soliman SS, Kim CN, Keefe MG, Hennick KM, Jain S, Li T, Tejera D, Shin D, Chhun BB, McGinnis CS, Speir M, Gartner ZJ, Mehta SB, Haeussler M, Hengen KB, Ransohoff RR, Piao X, Nowakowski TJ (2021). Human microglia states are conserved across experimental models and regulate neural stem cell responses in chimeric organoids. Cell Stem Cell.

[R212] Prinz M, Masuda T, Wheeler MA, Quintana FJ (2021). Microglia and central nervous system-associated macrophages-from origin to disease modulation. Annu Rev Immunol.

[R213] Priyathilaka TT, Laaker CJ, Herbath M, Fabry Z, Sandor M (2022). Modeling infectious diseases of the central nervous system with human brain organoids. Transl Res.

[R214] Qian X (2016). Brain-region-specific organoids using mini-bioreactors for modeling ZIKV exposure. Cell.

[R215] Qiao H, Deng X, Qiu L, Qu Y, Chiu Y, Chen F, Xia S, Muenzel C, Ge T, Zhang Z, Song P, Bonnin A, Zhao Z, Yuan W (2024). SARS-CoV-2 induces blood-brain barrier and choroid plexus barrier impairments and vascular inflammation in mice. J Med Virol.

[R216] Qiu L, Zhang B, Gao Z (2022). Lighting up neural circuits by viral tracing. Neurosci Bull.

[R217] Qu F (2023). Crosstalk between small-cell lung cancer cells and astrocytes mimics brain development to promote brain metastasis. Nat Cell Biol.

[R218] Quadrato G, Nguyen T, Macosko EZ, Sherwood JL, Min Yang S, Berger DR, Maria N, Scholvin J, Goldman M, Kinney JP, Boyden ES, Lichtman JW, Williams ZM, McCarroll SA, Arlotta P (2017). Cell diversity and network dynamics in photosensitive human brain organoids. Nature.

[R219] Rahimi Darehbagh R, Seyedoshohadaei SA, Ramezani R, Rezaei N (2024). Stem cell therapies for neurological disorders: current progress, challenges, and future perspectives. Eur J Med Res.

[R220] Reid JA, Mollica PA, Johnson GD, Ogle RC, Bruno RD, Sachs PC (2016). Accessible bioprinting: adaptation of a low-cost 3D-printer for precise cell placement and stem cell differentiation. Biofabrication.

[R221] Renner H, Grabos M, Becker KJ, Kagermeier TE, Wu J, Otto M, Peischard S, Zeuschner D, TsyTsyura Y, Disse P, Klingauf J, Leidel SA, Seebohm G, Schöler HR, Bruder JM (2020). A fully automated high-throughput workflow for 3D-based chemical screening in human midbrain organoids. Elife.

[R222] Reumann D, Krauditsch C, Novatchkova M, Sozzi E, Wong SN, Zabolocki M, Priouret M, Doleschall B, Ritzau-Reid KI, Piber M, Morassut I, Fieseler C, Fiorenzano A, Stevens MM, Zimmer M, Bardy C, Parmar M, Knoblich JA (2023). In vitro modeling of the human dopaminergic system using spatially arranged ventral midbrain-striatum-cortex assembloids. Nat Methods.

[R223] Revah O (2022). Maturation and circuit integration of transplanted human cortical organoids. Nature.

[R224] Reyes-Esteves S, Kumar M, Kasner SE, Witsch J (2023). Clinical grading scales and neuroprognostication in acute brain injury. Semin Neurol.

[R225] Rezaei B, Giacomoni J, Nilsson F, Sozzi E, Fiorenzano A, Parmar M, Keller SS, Kajtez J (2023). Modular 3D printed platform for fluidically connected human brain organoid culture. Biofabrication.

[R226] Rosety I, Zagare A, Saraiva C, Nickels S, Antony P, Almeida C, Glaab E, Halder R, Velychko S, Rauen T, Schöler HR, Bolognin S, Sauter T, Jarazo J, Krüger R, Schwamborn JC (2023). Impaired neuron differentiation in GBA-associated Parkinson’s disease is linked to cell cycle defects in organoids. NPJ Parkinsons Dis.

[R227] Roth JG, Brunel LG, Huang MS, Liu Y, Cai B, Sinha S, Yang F, Pașca SP, Shin S, Heilshorn SC (2023). Spatially controlled construction of assembloids using bioprinting. Nat Commun.

[R228] Rubio-Hernández EI, Comas-García M, Coronado-Ipiña MA, Colunga-Saucedo M, González Sánchez HM, Castillo CG (2023). Astrocytes derived from neural progenitor cells are susceptible to Zika virus infection. PLoS One.

[R229] Russo FB, Freitas BC, Pignatari GC, Fernandes IR, Sebat J, Muotri AR, Beltrão-Braga PCB (2018). Modeling the Interplay Between Neurons and Astrocytes in Autism Using Human Induced Pluripotent Stem Cells. Biol Psychiatry.

[R230] Rustenhoven J, Kipnis J (2022). Brain borders at the central stage of neuroimmunology. Nature.

[R231] Rybak-Wolf A, Wyler E, Pentimalli TM, Legnini I, Oliveras Martinez A, Glažar P, Loewa A, Kim SJ, Kaufer BB, Woehler A, Landthaler M, Rajewsky N (2023). Modelling viral encephalitis caused by herpes simplex virus 1 infection in cerebral organoids. Nat Microbiol.

[R232] Sabate-Soler S, Nickels SL, Saraiva C, Berger E, Dubonyte U, Barmpa K, Lan YJ, Kouno T, Jarazo J, Robertson G, Sharif J, Koseki H, Thome C, Shin JW, Cowley SA, Schwamborn JC (2022). Microglia integration into human midbrain organoids leads to increased neuronal maturation and functionality. Glia.

[R233] Sabogal-Guaqueta AM, Mitchell-Garcia T, Hunneman J, Voshart D, Thiruvalluvan A, Foijer F, Kruyt F, Trombetta-Lima M, Eggen BJL, Boddeke E, Barazzuol L, Dolga AM (2024). Brain organoid models for studying the function of iPSC-derived microglia in neurodegeneration and brain tumours. Neurobiol Dis.

[R234] Salg GA, Poisel E, Neulinger-Munoz M, Gerhardus J, Cebulla D, Bludszuweit-Philipp C, Vieira V, Nickel F, Herr I, Blaeser A, Giese NA, Hackert T, Kenngott HG (2022). Toward 3D-bioprinting of an endocrine pancreas: A building-block concept for bioartificial insulin-secreting tissue. J Tissue Eng.

[R235] Sali M, Carfì A, Di Paola A, Pereyra Boza M, Zampino G, Sanguinetti M, Landi F, Onder G (2022). SARS-CoV-2 vaccine humoral response in adults with Down syndrome. Clin Microbiol Infect.

[R236] Salick MR, Wells MF, Eggan K, Kaykas A (2017). Modelling Zika virus infection of the developing human brain in vitro using stem cell derived cerebral organoids. J Vis Exp.

[R237] Sandoval SO, Cappuccio G, Kruth K, Osenberg S, Khalil SM, Méndez-Albelo NM, Padmanabhan K, Wang D, Niciu MJ, Bhattacharyya A, Stein JL, Sousa AMM, Waxman EA, Buttermore ED, Whye D, Sirois CL, Williams A, Maletic-Savatic M, Zhao X (2024). Rigor and reproducibility in human brain organoid research: Where we are and where we need to go. Stem Cell Reports.

[R238] Sato T, Vries RG, Snippert HJ, van de Wetering M, Barker N, Stange DE, van Es JH, Abo A, Kujala P, Peters PJ, Clevers H (2009). Single Lgr5 stem cells build crypt-villus structures in vitro without a mesenchymal niche. Nature.

[R239] Sauerer T, Velázquez GF, Schmid C (2023). Relapse of acute myeloid leukemia after allogeneic stem cell transplantation: immune escape mechanisms and current implications for therapy. Mol Cancer.

[R240] Sawada T, Barbosa AR, Araujo B, McCord AE, D’Ignazio L, Benjamin KJM, Sheehan B, Zabolocki M, Feltrin A, Arora R, Brandtjen AC, Kleinman JE, Hyde TM, Bardy C, Weinberger DR, Paquola ACM, Erwin JA (2024). Recapitulation of perturbed striatal gene expression dynamics of donors’ brains with ventral forebrain organoids derived from the same individuals with schizophrenia. Am J Psychiatry.

[R241] Schafer ST, Mansour AA, Schlachetzki JCM, Pena M, Ghassemzadeh S, Mitchell L, Mar A, Quang D, Stumpf S, Ortiz IS, Lana AJ, Baek C, Zaghal R, Glass CK, Nimmerjahn A, Gage FH (2023). An in vivo neuroimmune organoid model to study human microglia phenotypes. Cell.

[R242] Schöbel A, Pinho Dos Reis V, Burkhard R, Hehner J, Schneider L, Schauflinger M, Vieyres G, Herker E (2024). Inhibition of sterol O-acyltransferase 1 blocks Zika virus infection in cell lines and cerebral organoids. Commun Biol.

[R243] Seth AK, Bayne T (2022). Theories of consciousness. Nat Rev Neurosci.

[R244] Shaji M, Tamada A, Fujimoto K, Muguruma K, Karsten SL, Yokokawa R (2024). Deciphering potential vascularization factors of on-chip co-cultured hiPSC-derived cerebral organoids. Lab Chip.

[R245] Shaker MR, Slonchak A, Al-Mhanawi B, Morrison SD, Sng JDJ, Cooper-White J, Khromykh AA, Wolvetang EJ (2024). Choroid plexus defects in Down syndrome brain organoids enhance neurotropism of SARS-CoV-2. Sci Adv.

[R246] Shakeri A, Wang Y, Zhao Y, Landau S, Perera K, Lee J, Radisic M (2023). Engineering organ-on-a-chip systems for vascular diseases. Arterioscler Thromb Vasc Biol.

[R247] Shan Y, Sun G, Ji J, Li Z, Chen X, Zhang X, Ma Y, Zhang Y, Zhang T, Zhang Y (2024). Brain function abnormalities and neuroinflammation in people living with HIV-associated anxiety disorders. Front Psychiatry.

[R248] Shannon JM, Mason RJ, Jennings SD (1987). Functional differentiation of alveolar type II epithelial cells in vitro: effects of cell shape, cell-matrix interactions and cell-cell interactions. Biochim Biophys Acta.

[R249] Shi Y, Sun L, Wang M, Liu J, Zhong S, Li R, Li P, Guo L, Fang A, Chen R, Ge WP, Wu Q, Wang X (2020). Vascularized human cortical organoids (vOrganoids) model cortical development in vivo. PLoS Biol.

[R250] Skardal A, Aleman J, Forsythe S, Rajan S, Murphy S, Devarasetty M, Pourhabibi Zarandi N, Nzou G, Wicks R, Sadri-Ardekani H, Bishop C, Soker S, Hall A, Shupe T, Atala A (2020). Drug compound screening in single and integrated multi-organoid body-on-a-chip systems. Biofabrication.

[R251] Smirnova L, Morales Pantoja IE, Hartung T (2023). Organoid intelligence (OI) - The ultimate functionality of a brain microphysiological system. Altex.

[R252] Song E (2021). Neuroinvasion of SARS-CoV-2 in human and mouse brain. J Exp Med.

[R253] Srivastava A, Nalroad Sundararaj S, Bhatia J, Singh Arya D (2024). Understanding long COVID myocarditis: A comprehensive review. Cytokine.

[R254] Stankovic I, Notaras M, Wolujewicz P, Lu T, Lis R, Ross ME, Colak D (2024). Schizophrenia endothelial cells exhibit higher permeability and altered angiogenesis patterns in patient-derived organoids. Transl Psychiatry.

[R255] Sun H (2024). Prediction of clinical precision chemotherapy by patient-derived 3D bioprinting models of colorectal cancer and its liver metastases. Adv Sci (Weinh).

[R256] Sun X, Kofman S, Ogbolu VC, Karch CM, Ibric L, Qiang L (2024). Vascularized brain assembloids with enhanced cellular complexity provide insights into the cellular deficits of tauopathy. Stem Cells.

[R257] Sun XY, Ju XC, Li Y, Zeng PM, Wu J, Zhou YY, Shen LB, Dong J, Chen YJ, Luo ZG (2022). Generation of vascularized brain organoids to study neurovascular interactions. Elife.

[R258] Sun Y, Jiang X, Gao J (2024). Stem cell-based ischemic stroke therapy: Novel modifications and clinical challenges. Asian J Pharm Sci.

[R259] Swingler M, Donadoni M, Bellizzi A, Cakir S, Sariyer IK (2023). iPSC-derived three-dimensional brain organoid models and neurotropic viral infections. J Neurovirol.

[R260] Tabrizi SJ, Estevez-Fraga C, van Roon-Mom WMC, Flower MD, Scahill RI, Wild EJ, Muñoz-Sanjuan I, Sampaio C, Rosser AE, Leavitt BR (2022). Potential disease-modifying therapies for Huntington’s disease: lessons learned and future opportunities. Lancet Neurol.

[R261] Tadokoro T (2024). Human iPSC-liver organoid transplantation reduces fibrosis through immunomodulation. Sci Transl Med.

[R262] Takebe T, Sekine K, Enomura M, Koike H, Kimura M, Ogaeri T, Zhang RR, Ueno Y, Zheng YW, Koike N, Aoyama S, Adachi Y, Taniguchi H (2013). Vascularized and functional human liver from an iPSC-derived organ bud transplant. Nature.

[R263] Tang XY, Xu L, Wang J, Hong Y, Wang Y, Zhu Q, Wang D, Zhang XY, Liu CY, Fang KH, Han X, Wang S, Wang X, Xu M, Bhattacharyya A, Guo X, Lin M, Liu Y (2021). DSCAM/PAK1 pathway suppression reverses neurogenesis deficits in iPSC-derived cerebral organoids from patients with Down syndrome. J Clin Invest.

[R264] Tang XY, Wu S, Wang D, Chu C, Hong Y, Tao M, Hu H, Xu M, Guo X, Liu Y (2022). Human organoids in basic research and clinical applications. Signal Transduct Target Ther.

[R265] Tebon PJ, Wang B, Markowitz AL, Davarifar A, Tsai BL, Krawczuk P, Gonzalez AE, Sartini S, Murray GF, Nguyen HTL, Tavanaie N, Nguyen TL, Boutros PC, Teitell MA, Soragni A (2023). Drug screening at single-organoid resolution via bioprinting and interferometry. Nat Commun.

[R266] Thion MS, Ginhoux F, Garel S (2018). Microglia and early brain development: An intimate journey. Science.

[R267] Thomas CA, Tejwani L, Trujillo CA, Negraes PD, Herai RH, Mesci P, Macia A, Crow YJ, Muotri AR (2017). Modeling of TREX1-dependent autoimmune disease using human stem cells highlights L1 accumulation as a source of neuroinflammation. Cell Stem Cell.

[R268] Thomson JA, Itskovitz-Eldor J, Shapiro SS, Waknitz MA, Swiergiel JJ, Marshall VS, Jones JM (1998). Embryonic stem cell lines derived from human blastocysts. Science.

[R269] Tian F (2022). Core transcription programs controlling injury-induced neurodegeneration of retinal ganglion cells. Neuron.

[R270] Tidball AM, Niu W, Ma Q, Takla TN, Walker JC, Margolis JL, Mojica-Perez SP, Sudyk R, Deng L, Moore SJ, Chopra R, Shakkottai VG, Murphy GG, Yuan Y, Isom LL, Li JZ, Parent JM (2023). Deriving early single-rosette brain organoids from human pluripotent stem cells. Stem Cell Reports.

[R271] Tomaskovic-Crook E, Higginbottom SL, Zhang B, Bourke J, Wallace GG, Crook JM (2023). Defined, simplified, scalable, and clinically compatible hydrogel-based production of human brain organoids. Organoids.

[R272] Trujillo CA, Gao R, Negraes PD, Gu J, Buchanan J, Preissl S, Wang A, Wu W, Haddad GG, Chaim IA, Domissy A, Vandenberghe M, Devor A, Yeo GW, Voytek B, Muotri AR (2019). Complex oscillatory waves emerging from cortical organoids model early human brain network development. Cell Stem Cell.

[R273] Tsuchida T, Murata S, Hasegawa S, Mikami S, Enosawa S, Hsu HC, Fukuda A, Okamoto S, Mori A, Matsuo M, Kawakatsu Y, Matsunari H, Nakano K, Nagashima H, Taniguchi H (2020). Investigation of clinical safety of human iPS cell-derived liver organoid transplantation to infantile patients in porcine model. Cell Transplant.

[R274] Tyagi K, Rai P, Gautam A, Kaur H, Kapoor S, Suttee A, Jaiswal PK, Sharma A, Singh G, Barnwal RP (2023). Neurological manifestations of SARS-CoV-2: complexity, mechanism and associated disorders. Eur J Med Res.

[R275] van Eyk CL, Fahey MC, Gecz J (2023). Redefining cerebral palsies as a diverse group of neurodevelopmental disorders with genetic aetiology. Nat Rev Neurol.

[R276] Vancamp P, Spirhanzlova P, Sébillot A, Butruille L, Gothié JD, Le Mével S, Leemans M, Wejaphikul K, Meima M, Mughal BB, Roques P, Remaud S, Fini JB, Demeneix BA (2021). The pyriproxyfen metabolite, 4’-OH-PPF, disrupts thyroid hormone signaling in neural stem cells, modifying neurodevelopmental genes affected by ZIKA virus infection. Environ Pollut.

[R277] Velasco S, Kedaigle AJ, Simmons SK, Nash A, Rocha M, Quadrato G, Paulsen B, Nguyen L, Adiconis X, Regev A, Levin JZ, Arlotta P (2019). Individual brain organoids reproducibly form cell diversity of the human cerebral cortex. Nature.

[R278] Velasco S (2022). Modeling brain disorders using transplanted organoids: Beyond the short circuit. Cell Stem Cell.

[R279] Villa CE, Cheroni C, Dotter CP, López-Tóbon A, Oliveira B, Sacco R, Yahya A, Morandell J, Gabriele M, Tavakoli MR, Lyudchik J, Sommer C, Gabitto M, Danzl JG, Testa G, Novarino G (2022). CHD8 haploinsufficiency links autism to transient alterations in excitatory and inhibitory trajectories. Cell Rep.

[R280] von Wrede R, Schidlowski M, Huppertz HJ, Rüber T, Ivo A, Baumgartner T, Hallmann K, Zsurka G, Helmstaedter C, Surges R, Kunz WS (2022). Large phenotypic variation of individuals from a family with a novel ASPM mutation associated with microcephaly, epilepsy, and behavioral and cognitive deficits. Genes (Basel).

[R281] Walsh RM, Luongo R, Giacomelli E, Ciceri G, Rittenhouse C, Verrillo A, Galimberti M, Bocchi VD, Wu Y, Xu N, Mosole S, Muller J, Vezzoli E, Jungverdorben J, Zhou T, Barker RA, Cattaneo E, Studer L, Baggiolini A (2024). Generation of human cerebral organoids with a structured outer subventricular zone. Cell Rep.

[R282] Wang C, Zhang M, Garcia G, Tian E, Cui Q, Chen X, Sun G, Wang J, Arumugaswami V, Shi Y (2021). ApoE-isoform-dependent SARS-CoV-2 neurotropism and cellular response. Cell Stem Cell.

[R283] Wang C, Sun M, Shao C, Schlicker L, Zhuo Y, Harim Y, Peng T, Tian W, Stöffler N, Schneider M, Helm D, Chu Y, Fu B, Jin X, Mallm JP, Mall M, Wu Y, Schulze A, Liu HK (2024). A multidimensional atlas of human glioblastoma-like organoids reveals highly coordinated molecular networks and effective drugs. NPJ Precis Oncol.

[R284] Wang F, Song P, Wang J, Wang S, Liu Y, Bai L, Su J (2024). Organoid bioinks: construction and application. Biofabrication.

[R285] Wang H, Matsushita MT (2021). Heavy metals and adult neurogenesis. Curr Opin Toxicol.

[R286] Wang H, Ning X, Zhao F, Zhao H, Li D (2024). Human organoids-on-chips for biomedical research and applications. Theranostics.

[R287] Wang H, Li J, Qin R, Guo F, Wang R, Bian Y, Chen H, Yuan H, Pan Y, Jin J, Wang Y, Du Y, Wu F (2024). Porous gelatin methacrylate gel engineered by freeze-ultraviolet promotes osteogenesis and angiogenesis. ACS Biomater Sci Eng.

[R288] Wang L, Li Z, Sievert D, Smith DEC, Mendes MI, Chen DY, Stanley V, Ghosh S, Wang Y, Kara M, Aslanger AD, Rosti RO, Houlden H, Salomons GS, Gleeson JG (2020). Loss of NARS1 impairs progenitor proliferation in cortical brain organoids and leads to microcephaly. Nat Commun.

[R289] Wang L, Sievert D, Clark AE, Lee S, Federman H, Gastfriend BD, Shusta EV, Palecek SP, Carlin AF, Gleeson JG (2021). A human three-dimensional neural-perivascular ‘assembloid’ promotes astrocytic development and enables modeling of SARS-CoV-2 neuropathology. Nat Med.

[R290] Wang LL, Serrano C, Zhong X, Ma S, Zou Y, Zhang CL (2021). Revisiting astrocyte to neuron conversion with lineage tracing in vivo. Cell.

[R291] Wang M, Zhang L, Novak SW, Yu J, Gallina IS, Xu LL, Lim CK, Fernandes S, Shokhirev MN, Williams AE, Saxena MD, Coorapati S, Parylak SL, Quintero C, Molina E, Andrade LR, Manor U, Gage FH (2025). Morphological diversification and functional maturation of human astrocytes in glia-enriched cortical organoid transplanted in mouse brain. Nat Biotechnol.

[R292] Wang P, Jin L, Zhang M, Wu Y, Duan Z, Guo Y, Wang C, Guo Y, Chen W, Liao Z, Wang Y, Lai R, Lee LP, Qin J (2024). Blood-brain barrier injury and neuroinflammation induced by SARS-CoV-2 in a lung-brain microphysiological system. Nat Biomed Eng.

[R293] Wang Q, Dong X, Hu T, Qu C, Lu J, Zhou Y, Li J, Pei G (2021). Constitutive activity of serotonin receptor 6 regulates human cerebral organoids formation and depression-like behaviors. Stem Cell Reports.

[R294] Wang Q, Yang Q, Liu X (2023). The microbiota-gut-brain axis and neurodevelopmental disorders. Protein Cell.

[R295] Wang Q, Wang M, Choi I, Sarrafha L, Liang M, Ho L, Farrell K, Beaumont KG, Sebra R, De Sanctis C, Crary JF, Ahfeldt T, Blanchard J, Neavin D, Powell J, Davis DA, Sun X, Zhang B, Yue Z (2024). Molecular profiling of human substantia nigra identifies diverse neuron types associated with vulnerability in Parkinson’s disease. Sci Adv.

[R296] Wang SN, Wang Z, Xu TY, Cheng MH, Li WL, Miao CY (2020). Cerebral organoids repair ischemic stroke brain injury. Transl Stroke Res.

[R297] Wang Z, Wang SN, Xu TY, Hong C, Cheng MH, Zhu PX, Lin JS, Su DF, Miao CY (2020). Cerebral organoids transplantation improves neurological motor function in rat brain injury. CNS Neurosci Ther.

[R298] Watanabe K, Ueno M, Kamiya D, Nishiyama A, Matsumura M, Wataya T, Takahashi JB, Nishikawa S, Nishikawa S, Muguruma K, Sasai Y (2007). A ROCK inhibitor permits survival of dissociated human embryonic stem cells. Nat Biotechnol.

[R299] Watanabe S, Kobayashi S, Ogasawara N, Okamoto R, Nakamura T, Watanabe M, Jensen KB, Yui S (2022). Transplantation of intestinal organoids into a mouse model of colitis. Nat Protoc.

[R300] Watari K (2023). Self-organization, quality control, and preclinical studies of human iPSC-derived retinal sheets for tissue-transplantation therapy. Commun Biol.

[R301] Weiss P, Taylor AC (1960). REconstitution of complete organs from single-cell suspensions of chick embryos in advanced stages of differentiation. Proc Natl Acad Sci U S A.

[R302] Werschler N, Penninger J (2023). Generation of human blood vessel organoids from pluripotent stem cells. J Vis Exp.

[R303] Wilson HV (1907). A new method by which sponges may be artificially reared. Science.

[R304] Wilson MN, Thunemann M, Liu X, Lu Y, Puppo F, Adams JW, Kim JH, Ramezani M, Pizzo DP, Djurovic S, Andreassen OA, Mansour AA, Gage FH, Muotri AR, Devor A, Kuzum D (2022). Multimodal monitoring of human cortical organoids implanted in mice reveal functional connection with visual cortex. Nat Commun.

[R305] Wongsawat J (2024). Characteristics, risk factors, and outcomes related to Zika virus infection during pregnancy in Northeastern Thailand: A prospective pregnancy cohort study, 2018–2020. PLoS Negl Trop Dis.

[R306] Woods CG (2004). Human microcephaly. Curr Opin Neurobiol.

[R307] Wu CT (2023). SARS-CoV-2 replication in airway epithelia requires motile cilia and microvillar reprogramming. Cell.

[R308] Wu J (2024). Microglial over-pruning of synapses during development in autism-associated SCN2A-deficient mice and human cerebral organoids. Mol Psychiatry.

[R309] Wu S, Hong Y, Chu C, Gan Y, Li X, Tao M, Wang D, Hu H, Zheng Z, Zhu Q, Han X, Zhu W, Xu M, Dong Y, Liu Y, Guo X (2024). Construction of human 3D striato-nigral assembloids to recapitulate medium spiny neuronal projection defects in Huntington’s disease. Proc Natl Acad Sci U S A.

[R310] Wu X, Xu S, Wang P, Wang ZQ, Chen H, Xu X, Peng B (2022). ASPM promotes ATR-CHK1 activation and stabilizes stalled replication forks in response to replication stress. Proc Natl Acad Sci U S A.

[R311] Wu Y, Qin M, Yang X (2023). Organ bioprinting: progress, challenges and outlook. J Mater Chem B.

[R312] Wulansari N, Darsono WHW, Woo HJ, Chang MY, Kim J, Bae EJ, Sun W, Lee JH, Cho IJ, Shin H, Lee SJ, Lee SH (2021). Neurodevelopmental defects and neurodegenerative phenotypes in human brain organoids carrying Parkinson’s disease-linked DNAJC6 mutations. Sci Adv.

[R313] Xie X, Wang L, Dong S, Ge S, Zhu T (2024). Immune regulation of the gut-brain axis and lung-brain axis involved in ischemic stroke. Neural Regen Res.

[R314] Xu C, Yuan X, Hou P, Li Z, Wang C, Fang C, Tan Y (2023). Development of glioblastoma organoids and their applications in personalized therapy. Cancer Biol Med.

[R315] Xu J, Fang S, Deng S, Li H, Lin X, Huang Y, Chung S, Shu Y, Shao Z (2023). Generation of neural organoids for spinal-cord regeneration via the direct reprogramming of human astrocytes. Nat Biomed Eng.

[R316] Xu L, Huo HQ, Lu KQ, Tang XY, Hong Y, Han X, Fu ZX, Fang KH, Xu M, Guo X, Liu Y (2022). Abnormal mitochondria in Down syndrome iPSC-derived GABAergic interneurons and organoids. Biochim Biophys Acta Mol Basis Dis.

[R317] Xu L, Ding H, Wu S, Xiong N, Hong Y, Zhu W, Chen X, Han X, Tao M, Wang Y, Wang D, Xu M, Huo D, Gu Z, Liu Y (2024). Artificial Meshed Vessel-Induced Dimensional Breaking Growth of Human Brain Organoids and Multiregional Assembloids. ACS nano.

[R318] Xu M (2016). Identification of small-molecule inhibitors of Zika virus infection and induced neural cell death via a drug repurposing screen. Nat Med.

[R319] Xu R, Brawner AT, Li S, Liu JJ, Kim H, Xue H, Pang ZP, Kim WY, Hart RP, Liu Y, Jiang P (2019). OLIG2 drives abnormal neurodevelopmental phenotypes in human iPSC-based organoid and chimeric mouse models of Down syndrome. Cell Stem Cell.

[R320] Xu X, Gao W, Cheng S, Yin D, Li F, Wu Y, Sun D, Zhou S, Wang D, Zhang Y, Jiang R, Zhang J (2017). Anti-inflammatory and immunomodulatory mechanisms of atorvastatin in a murine model of traumatic brain injury. J Neuroinflammation.

[R321] Xu YP (2019). Zika virus infection induces RNAi-mediated antiviral immunity in human neural progenitors and brain organoids. Cell Res.

[R322] Xue X, Kim YS, Ponce-Arias AI, O’Laughlin R, Yan RZ, Kobayashi N, Tshuva RY, Tsai YH, Sun S, Zheng Y, Liu Y, Wong FCK, Surani A, Spence JR, Song H, Ming GL, Reiner O, Fu J (2024). A patterned human neural tube model using microfluidic gradients. Nature.

[R323] Yakoub AM, Sadek M (2018). Development and characterization of human cerebral organoids: An optimized protocol. Cell Transplant.

[R324] Yamagami K, Samata B, Doi D, Tsuchimochi R, Kikuchi T, Amimoto N, Ikeda M, Yoshimoto K, Takahashi J (2024). Progranulin enhances the engraftment of transplanted human iPS cell-derived cerebral neurons. Stem Cells Transl Med.

[R325] Yan Y, Li X, Gao Y, Mathivanan S, Kong L, Tao Y, Dong Y, Li X, Bhattacharyya A, Zhao X, Zhang SC (2024). 3D bioprinting of human neural tissues with functional connectivity. Cell Stem Cell.

[R326] Yang M, Bai M, Zhuang Y, Lu S, Ge Q, Li H, Deng Y, Wu H, Xu X, Niu F, Dong X, Zhang B, Liu B (2025). High-dose dexamethasone regulates microglial polarization via the GR/JAK1/STAT3 signaling pathway after traumatic brain injury. Neural Regen Res.

[R327] Yin K, Wang D, Zhao H, Wang Y, Zhang Y, Liu Y, Li B, Xing M (2022). Polystyrene microplastics up-regulates liver glutamine and glutamate synthesis and promotes autophagy-dependent ferroptosis and apoptosis in the cerebellum through the liver-brain axis. Environ Pollut.

[R328] Zamponi M, Mollica PA, Khodour Y, Bjerring JS, Bruno RD, Sachs PC (2023). Combined 3D bioprinting and tissue-specific ECM system reveals the influence of brain matrix on stem cell differentiation. Front Cell Dev Biol.

[R329] Zeng X, Ma Q, Li XK, You LT, Li J, Fu X, You FM, Ren YF (2023). Patient-derived organoids of lung cancer based on organoids-on-a-chip: enhancing clinical and translational applications. Front Bioeng Biotechnol.

[R330] Zhang FL, Hu Z, Wang YF, Zhang WJ, Zhou BW, Sun QS, Lin ZB, Liu KX (2023). Organoids transplantation attenuates intestinal ischemia/reperfusion injury in mice through L-Malic acid-mediated M2 macrophage polarization. Nat Commun.

[R331] Zhang S, Li N, Wu S, Xie T, Chen Q, Wu J, Zeng S, Zhu L, Bai S, Zha H, Tian W, Wu N, Zou X, Fang S, Luo C, Shi M, Sun C, Shu Y, Luo H (2024). c-FLIP facilitates ZIKV infection by mediating caspase-8/3-dependent apoptosis. PLoS Pathog.

[R332] Zhang Y, Liu J, Huang L, Wang Z, Wang L (2015). Design and performance of a sericin-alginate interpenetrating network hydrogel for cell and drug delivery. Sci Rep.

[R333] Zhang Y, Chen H, Long X, Xu T (2022). Three-dimensional-engineered bioprinted in vitro human neural stem cell self-assembling culture model constructs of Alzheimer’s disease. Bioact Mater.

[R334] Zhang Y, Tang C, He Y, Zhang Y, Li Q, Zhang T, Zhao B, Tong A, Zhong Q, Zhong Z (2024). Semaglutide ameliorates Alzheimer’s disease and restores oxytocin in APP/PS1 mice and human brain organoid models. Biomed Pharmacother.

[R335] Zhao J (2020). APOE4 exacerbates synapse loss and neurodegeneration in Alzheimer’s disease patient iPSC-derived cerebral organoids. Nat Commun.

[R336] Zhao Y, Li S, Zhu L, Huang M, Xie Y, Song X, Chen Z, Lau HC, Sung JJ, Xu L, Yu J, Li X (2024). Personalized drug screening using patient-derived organoid and its clinical relevance in gastric cancer. Cell Rep Med.

[R337] Zeng M, Ding J, Tian Y, Zhang Y, Liu X, Chen Z, Sun J, Wu C, Yin H, Wei D, Fan H (2024). Dopamine-integrated all-hydrogel multi-electrode arrays for neural activity recording. Mater Horiz.

[R338] Zhong X, Harris G, Smirnova L, Zufferey V, Sá R, Baldino Russo F, Baleeiro Beltrao Braga PC, Chesnut M, Zurich MG, Hogberg HT, Hartung T, Pamies D (2020). Antidepressant paroxetine exerts developmental neurotoxicity in an iPSC-derived 3D human brain model. Front Cell Neurosci.

[R339] Zhou C, Wu Y, Wang Z, Liu Y, Yu J, Wang W, Chen S, Wu W, Wang J, Qian G, He A (2023). Standardization of organoid culture in cancer research. Cancer Med.

[R340] Zhou LF, Liao HY, Han Y, Zhao Y (2024). The use of organoids in creating immune microenvironments and treating gynecological tumors. J Transl Med.

[R341] Zhou LT (2023). Tau pathology epigenetically remodels the neuron-glial cross-talk in Alzheimer’s disease. Sci Adv.

[R342] Zhou T, Tan L, Cederquist GY, Fan Y, Hartley BJ, Mukherjee S, Tomishima M, Brennand KJ, Zhang Q, Schwartz RE, Evans T, Studer L, Chen S (2017). High-content screening in hPSC-neural progenitors identifies drug candidates that inhibit Zika virus infection in fetal-like organoids and adult brain. Cell Stem Cell.

[R343] Zhu S, Diao S, Liu X, Zhang Z, Liu F, Chen W, Lu X, Luo H, Cheng X, Liao Q, Li Z, Chen J (2025). Biomaterial-based strategies: a new era in spinal cord injury treatment. Neural Regen Res.

[R344] Zhu Y, Jiang D, Qiu Y, Liu X, Bian Y, Tian S, Wang X, Hsia KJ, Wan H, Zhuang L, Wang P (2024). Dynamic microphysiological system chip platform for high-throughput, customizable, and multi-dimensional drug screening. Bioact Mater.

